# Circulating RNAs as predictive markers for the progression of type 2 diabetes

**DOI:** 10.1111/jcmm.14182

**Published:** 2019-02-07

**Authors:** Vikas Ghai, David Baxter, Xiaogang Wu, Taek‐Kyun Kim, Johanna Kuusisto, Markku Laakso, Tom Connolly, Yong Li, Patricia Andrade‐Gordon, Kai Wang

**Affiliations:** ^1^ Institute for Systems Biology Seattle Washington; ^2^ Institute of Clinical Medicine Kuopio University Hospital, University of Eastern Finland Kuopio Finland; ^3^ Cardiovascular and Metabolism Therapeutic Area Janssen Research & Development Pennsylvania

**Keywords:** biomarker, diabetes, microRNAs, obesity, prediabetes

## Abstract

Type 2 Diabetes Mellitus (T2DM) is the most prevalent form of diabetes in the USA, thus, the identification of biomarkers that could be used to predict the progression from prediabetes to T2DM would be greatly beneficial. Recently, circulating RNA including microRNAs (miRNAs) present in various body fluids have emerged as potential biomarkers for various health conditions, including T2DM. Whereas studies that examine the changes of miRNA spectra between healthy controls and T2DM individuals have been reported, the goal of this study is to conduct a baseline comparison of prediabetic individuals who either progress to T2DM, or remain prediabetic. Using an advanced small RNA sequencing library construction method that improves the detection of miRNA species, we identified 57 miRNAs that showed significant concentration differences between progressors (progress from prediabetes to T2DM) and non‐progressors. Among them, 26 have been previously reported to be associated with T2DM in either body fluids or tissue samples. Some of the miRNAs identified were also affected by obesity. Furthermore, we identified miRNA panels that are able to discriminate progressors from non‐progressors. These results suggest that upon further validation these miRNAs may be useful to predict the risk of conversion to T2DM from prediabetes.

## 1 INTRODUCTION

With over 400 million cases worldwide, diabetes accounts for 5%‐20% of total health expenditures for most countries.^1^ In the United States alone, there are 29 million patients living with diabetes and 90%‐95% of these are cases of Type 2 Diabetes Mellitus (T2DM).^2^T2DM is defined by elevated blood glucose levels driven by insulin resistance (IR), followed by β‐cell dysfunction. Onset of T2DM is usually preceded by a prediabetic phase, classified as impaired fasting glucose (IFG), where blood glucose levels are elevated but below the indicative level for T2DM, impaired glucose tolerance (IGT), or elevated HbA1c. Currently, there are 84 million people in the US who are prediabetic, and it is estimated that up to 70% of these individuals will eventually progress to T2DM.^3^, ^4^, ^5^ There are currently several approaches that have been employed to halt or reverse the progression toward T2DM in prediabetic individuals, including reducing sugar intake, exercise and weight loss, and the use of glucose lowering drugs like Metformin.^6^, ^7^, ^8^ While glycated hemoglobin (HbA1c) and fasting plasma glucose (FPG) levels are widely used by clinicians to assess the progression and development of diabetes, they cannot be used to determine the risk of developing T2DM for a prediabetic individual.

Recently, circulating extracellular RNAs, especially microRNAs (miRNAs), present in various body fluids have emerged as potential biomarkers for various diseases and health conditions, including metabolic diseases such as diabetes and obesity.^9^ While many miRNAs show promise as biomarkers to distinguish patients with T2DM from healthy individuals, meta‐analysis examining specific miRNA concentration differences in plasma between T2DM and healthy individuals show inconsistencies among studies.^10^ Many of these discrepancies can be attributed to sample preparation differences and technical issues inherent to various miRNA measurement methods. These factors make the development of circulating miRNA‐based biomarkers for T2DM challenging. While qRT‐PCR is very well suited for measuring and validating the concentration changes of specific miRNAs especially in clinical setting, small‐RNA sequencing (sRNAseq) provides higher dynamic range than qRT‐PCR and offers better discrimination between closely related family members, making it a better method for discovery‐based miRNA studies. There are several commercial sRNAseq library construction kits available; however, they are prone to introducing significant bias, most likely occurring during adapter‐RNA ligation steps.^11^, ^12^ Such biases result in an inaccurate miRNA profile and can cause difficulties when validating the findings. Additionally, aligning and mapping miRNA can be a challenge due to their small size, and requires a specialized data analysis pipeline

To address issues associated with sRNAseq, we adapted a modified small RNA sequencing library construction method that incorporates four degenerate bases at the appropriate ends of adapters to facilitate the RNA‐adapter ligations. This reduces ligation associated bias and provides results that correlate better with qRT‐PCR.^13^, ^14^, ^15^ We have also developed a data analysis pipeline, sRNAnalyzer,^16^ which allows for more accurate mapping of small RNA sequencing results. Using these improved methods, we analyzed the spectrum of small RNA from plasma obtained from prediabetic individuals who participated in the METSIM (METabolic Syndrome In Men) study.^17^ From the baseline samples analyzed, half of the individuals progressed to T2DM, and the other half remained prediabetic, determined at 5‐year follow‐up. We identified several circulating extracellular miRNAs that are prognostic at baseline for the transition from prediabetes to T2DM. Some of these miRNAs were also affected by body mass index (BMI), with obese progressors showing greater concentration changes than progressors with a normal BMI. This is the first reported study of this type to look at predictive biomarkers of T2DM years before onset.

## 2 MATERIALS AND METHODS

### 2.1 Sample and study collection

Patient plasma samples were obtained from the METSIM (METabolic Syndrome In Men) study collection. METSIM was a population‐based study conducted in Finland from 2005 to 2010 to identify risk factors that would contribute to T2DM and cardiovascular disease in men.^17^ Male subjects, aged between 45 and 70 years, were randomly selected from the population register of the town of Kuopio in eastern Finland. Participants had a 1‐day outpatient visit to the Clinical Research Unit at the University of Kuopio, including an interview on the history of previous diseases and current drug treatment and an evaluation of glucose tolerance and cardiovascular risk factors. Fasting blood samples were drawn after 12 hours of fasting followed by an OGTT. Glucose tolerance was evaluated based on OGTT as follows: NGT (fasting plasma glucose [FPG] <5.6 mmol/L), isolated IFG (FPG 5.6‐6.9 mmol/L), and newly diagnosed type 2 diabetic subjects (FPG ≥7.0 mmol/L). The study was approved by the ethics committee of the University of Kuopio and Kuopio University Hospital, and it was in accordance with the Helsinki Declaration. The sample set represents 290 prediabetic individuals with isolated impaired fasting glucose (IsolIFG) that either progressed to T2DM (n = 145), or remained prediabetic (n = 145) after a five year follow up. Samples were controlled for age, body mass index (BMI), and FPG (Table 1, Supplemental Table S1) between progressors and non‐progressors. The plasma samples were processed as follows: Blood was collected into EDTA‐treated tubes, and spun at 1000 *g* at 4°C for 10 minutes to remove blood cells, and then 2000 *g* at 4°C for 15 minutes to remove platelets. Before RNA isolation, the plasma samples were spun at 10 000 *g* at 4°C for 10 minutes to remove any remaining cellular debris and platelets.

**Table 1:** Patient information and general mapping results

Category	All samples (mean)	Non‐progressor (mean)	Progressor (mean)	*P*‐value
General information				
Age	59.3	59.1	59.5	0.56721
BMI	28.2	28.2	28.2	0.96731
Fasting Plasma Glucose	6.15	6.08	6.22	0.00066
Raw read count	12 425 482	12 342 151	12 508 813	0.85227
Processed read	5 532 786	5 385 787	5 679 785	0.64124
Read mapped to human	2 336 167	2 191 826	2 480 508	0.42674
Number of reads mapped to different categories				
miRNA	1 590 537	1 509 478	1 671 596	0.51564
piRNA	84 836	70 885	98 787	0.16150
snoRNA	4 740	3 933	5 547	0.00639
LncRNA	12 421	10 418	14 424	0.02377
mRNA	43 284	38 850	47 717	0.05751
rRNA/tRNA	547 074	507 673	586 475	0.42710
Others	53 276	50 589	55 963	0.55875

*P*‐values <0.05 are underlined.

### 2.2 miRNA isolation and library construction

Circulating RNA was isolated from 75 µL of frozen plasma using the miRNeasy kit (QAIGEN, Germantown, MD) according to the manufacturer's instructions. The RNA was eluted in nuclease‐free H_2_0, and the quantity and quality were assessed using a Bioanalyzer (Agilent Technologies, Santa Clara, CA). To profile miRNA in plasma, we used a modified small‐RNA library construction protocol. Briefly, the method utilizes adapters containing four degenerate nucleotides at proper ends to enhance the adapter‐miRNA ligation and reduce ligation associated bias (3′ adapter sequence: /5rApp/(N:25252525)(N)(N)(N)TGGAATTCTCGGGTGCCAAGG/3ddC/; 5′ adapter sequence: rGrUrUrCrArGrArGrUrUrCrUrArCrArGrUrCrCrGrArCrGrArUrCr(N:25252525)r(N)r(N)r(N)).^13^ After adapter ligation and cDNA synthesis, the library was amplified for four cycles followed by an initial size‐selection of library inserts in the range between 127 bp and 156 bp on a Pippin HT automated size‐selection instrument (Sage Science, Beverly, MA). The purified fragments were then amplified for an additional 16 cycles, and then size selected again. This two‐step size selection significantly reduces the adapter dimer in the library. Individual sRNAseq (small RNA sequencing) library concentrations were assessed by NEBNext Library Quant Kit for Illumina (New England Biolabs, Ipswich, MA), pooled (2 nmol/L final concentration) and then run on a NextSeq500 sequencer (Illumina, San Diego, CA).

### 2.3 Data analysis

Sequence files were processed with an in‐house small RNA analysis pipeline—sRNAnalyzer.^16^ Briefly, the adapters were trimmed from the sequence reads, and low complexity (homo‐polymer and simple repeat sequences), low quality and short reads (less than 15 nucleotides) were removed from the file. The processed reads were then searched against various sequence databases. For miRNA, the reads were mapped against miRBase (www.mirbase.org). Data analysis was based on mapping results with 0 mis‐match allowed. The miRNA mapping data were normalized using read count per million of processed read and log2 transformed. Based on the results, several invariant miRNAs, including miR‐21‐5p, were identified. The miRTar database (*mirtarbase.mbc.nctu.edu.tw/*) was used to identify validated miRNA targets for gene enrichment analysis to identify biological processes that may be regulated by miRNA. In this approach we required that each miRNA target must be validated by at least two different techniques. The gene enrichment analysis was performed with DAVID (Database for Annotation, Visualization and Integrated Discovery, https://david.ncifcrf.gov/).

### 2.4 Novel miRNA analysis

After mapping against the miRNA database, the remaining unmapped reads from samples were combined and run through mirdeep2^18^ to identify putative miRNAs. A novel miRNA database was then built and integrated into sRNanalyzer. Unmapped reads from individual samples were then run against the novel miRNA database to determine the number of miRNA candidates in each sample.

### 2.5 qRT‐PCR

Quantitative Reverse Transcription Polymerase Chain Reaction (qRT‐PCR) validation of miRNAs was performed using TaqMan Advanced miRNA assays (Thermo Fisher, Waltham, MA). MiR‐21‐5p and miR‐16‐5p were used as a normalizers, since they were identified as an invariant miRNAs (low coefficient of variance across samples) based on the miRNA mapping results, and did not show concentration changes between progressors and non‐progressors. Relative miRNA values are presented as 40 (maximum cycle number) ‐ ΔΔCt values (Ct _reference _– Ct _target_) between progressors and non‐progressors (ΔCt _progressors ‐ _ΔCt _non‐progressors_.^14^, ^19^ Ct values greater than 35 were not considered as being expressed and were not used.

### 2.6 Identification of predictive miRNA panel

To examine the discrimination power of miRNAs, a classification score was calculated for each sample based on selected miRNAs. The geometric means of expression levels of the selected miRNAs were used as the classification score. The miRNA sets with the highest discrimination power were identified using greedy search algorithm.^20^ In brief, the approach started with the miRNA with the most significant discrimination ability, and then the remaining miRNAs were added one at a time to compute the classification score. Using the score, we calculated the area under the curve (AUC) value from a receiver operating characteristic (ROC) curve. The set of miRNAs showing the highest value of AUC were chosen as the classification panel. To evaluate the classification/prediction performance, we tested the miRNA panels using either all study participants, obese participants, or participants with normal BMI.

### 2.7 Statistical analysis

The Wilcoxon rank sum statistical test was used to determine the p‐value for miRNAs showing concentration differences between progressors and non‐progressors in both small‐RNAseq and qRT‐PCR. For multiple hypothesis correction, Benjamini‐Hochberg was used to identify FDR‐corrected *P *< 0.05, Multivariate analysis of covariance (MANCOVA) was used to identify miRNAs affected by significant differences in FPG between progressors and non‐progressors.

## 3 RESULTS

### 3.1 Sequencing results

From the 290 prediabetic individuals (Supplemental Table S1) that were selected from the METSIM study, 145 of them progressed to T2DM after five years (termed “progressors”) and 145 remained at the prediabetic stage (termed “non‐progressors”). The samples were matched with respect to BMI and age, but there was a significant difference in the fasting plasma glucose level between the two groups (Table 1). To explore the possible changes in extracellular small RNA profiles between the two groups, we isolated RNA (Supplementary Figure S1) characterized the plasma RNA using next generation sequencing. In summary, we obtained about 12 million reads on average across all samples, of which approximately 5.5 million reads (“processed” reads) passed the pre‐processing step, which removed adapter sequences and short or low complexity reads. There was no significant difference between the progressor and non‐progressor groups in sequencing read count. About 2.3 million processed reads mapped to human, among them 1.6 million reads mapped to various human miRNAs, while the remaining reads mapped to other RNA species, such as piRNA, snoRNA, rRNA/tRNA, lncRNA, and mRNA fragments. With the exception of snoRNA and lncRNA, there were no significant differences in the number of reads mapping to different classes of RNAs such as miRNA or piRNA (Table 1). About 700 different miRNAs were observed (with at least one mapped read) in each sample, among them about 500 miRNAs had 10 or more mapped reads (Table 1).

### 3.2 miRNAs associated with disease progression

**Figure 1:**
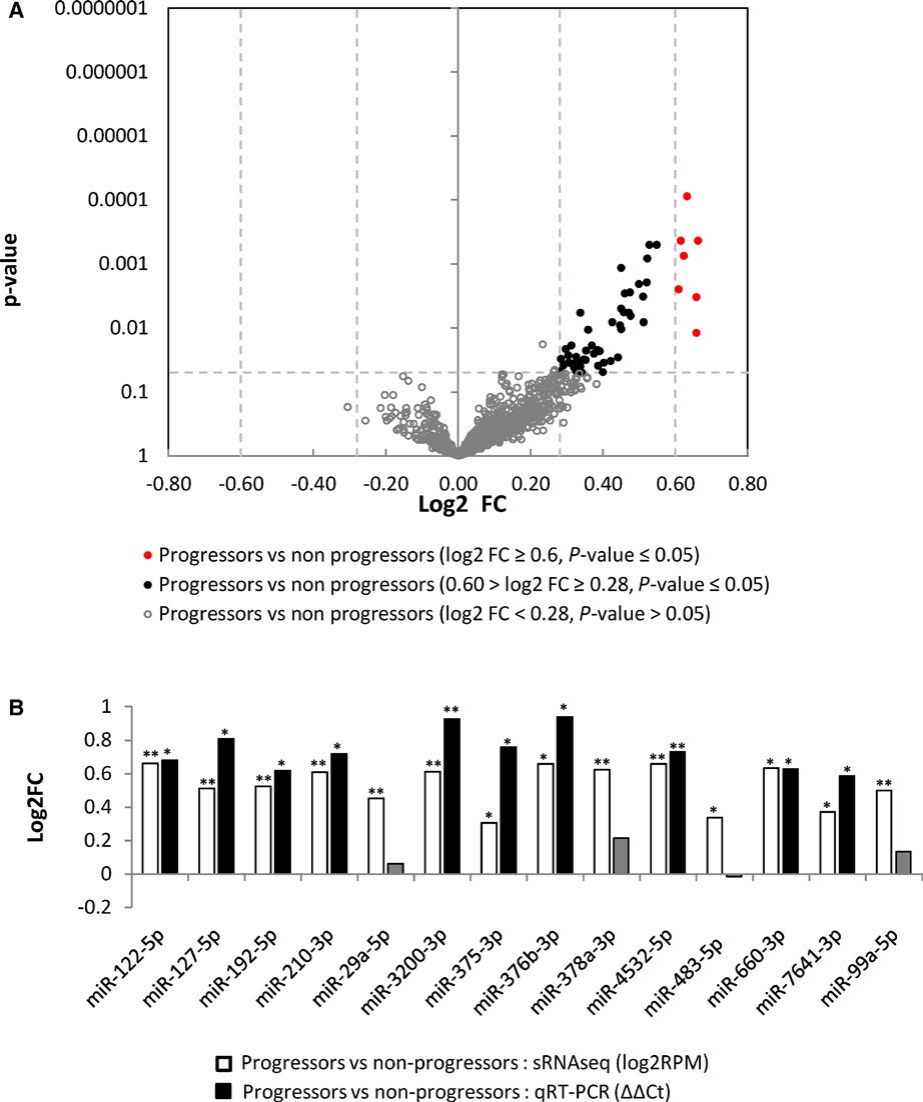
miRNAs showing concentration differences between progressors and non‐progressors. A, Volcano plot of log2 fold change (log2FC) vs *P*‐value between progressors and non‐progressors. Grey unfilled circles represent miRNAs with a *P* > 0.05, black circles represent miRNAs with a *P* < 0.05, and a Log2FC between ±0.28 and ±0.58 (Fold change between 1.2 and 1.5, respectively), and red circles represent miRNAs with a *P* < 0.05, and a log2FC>±0.58 (fold change greater than 1.5). B, qRT‐PCR results for 14 selected miRNAs (normalized to the average of miR‐16‐5p and miR‐21‐5p) between progressors and non‐progressors. Values are represented as log2FC (Y‐axis) and the miRNA identity is indicated on the X‐axis. The qRT‐PCR data (black bars) is derived from ΔΔCt values and the sRNAseq data (white bars) is derived from log2RPM values. Single asterisks indicate a *P* < 0.05, double asterisks indicate a *P* < 0.01 Grayed out values without an asterisks indicate an insignificant result

The Wilcoxon rank sum statistical test was used to identify circulating miRNAs that showed statistically significant concentration differences between progressors and non‐progressors. Using >1.5 fold concentration change and *P* < 0.05 as cut‐off, we identified 8 dysregulated miRNAs (miR‐122‐5p, miR‐210‐3p, miR‐3200‐3p, miR‐376b‐3p, miR378a‐3p, miR‐4532‐5p, miR‐483‐5p and miR‐660‐3p) all showing increased concentration in plasma samples from progressors compared to non‐progressors (Figure 1A: Red circles, Table 2). Some of the well‐known T2DM‐associated miRNAs such as miR‐375, miR‐192‐5p, and miR‐127‐5p showed statistically significant concentration differences between the groups but did not show >1.5 fold concentration changes. We therefore used *P* < 0.05 as cut‐off and fold change >1.2, and identified a total of 57 affected miRNAs (Figure 1A: Black circles, Table 2). To determine if the slight (though significant) difference in FPG between progressors and non‐progressors impacted any of these miRNAs, we performed MANCOVA analysis and identified nine of these 57 miRNAs that were affected by FPG (Supplemental Table S2), we excluded them for further analysis. From the 48 miRNAs associated with T2DM progression, we selected several (miR‐122‐5p, miR‐127‐5p, miR‐192‐5p, miR‐210‐3p, miR‐29a‐5p, miR‐3200‐3p, miR‐375‐3p, miR‐376b‐3p, miR‐378a‐3p, miR‐4532‐5p, miR‐483‐5p, and miR‐660‐3p, miR‐7641‐3p, and miR‐99a‐5p) for qRT‐PCR verification on all 290 samples. These were chosen based on an observed fold change of 1.5 or higher (miR‐122‐5p, miR‐210‐3p, miR‐3200‐3p, and miR‐376b‐3p, miR‐378a‐3p, miR‐4532‐5p, and miR‐483‐5p, miR‐660‐3p), or a reported association with T2DM but with fold changes between 1.2 and 1.5 fold (miR‐127‐5p, miR‐192‐5p, miR‐29a‐5p, miR‐375‐3p, miR‐99a‐5p). The fold changes and statistical significance of most of miRNAs do not change with different normalization approaches (Supplementary Figure S2). The qRT‐PCR results confirmed elevated levels of several of these miRNAs, including miR‐122‐5p, miR‐127‐5p, miR‐192‐5p, miR‐210‐3p, miR‐3200‐3p, miR‐375‐3p, miR‐376b‐3p, miR‐4532‐5p, and miR‐660‐3p, and miR‐7641‐3p (Figure 1B).

**Table 2:** List of miRNAs showing concentration changes in the study

miRNA					Obese (BMI >30) vs normal (BMI <25)						Progressors vs non‐progressors					
	Progressors vs non‐progressors				Progressors			non‐progressors			Normal (BMI <25)			Obese (BMI >30)		
					PO vs PN			NPO vs NPN			PN vs NPN			PO vs NPO		
	Log 2 FC (> 1.5 fold change)	Log 2 FC (> 1.2 fold change)	*P*‐value	B‐H FDR	Log 2 FC (> 1.5 fold change	*P*‐value	B‐H FDR	Log 2 FC (> 1.5 fold change	*P*‐value	B‐H FDR	Log 2 FC (> 1.5 fold change	*P*‐value	B‐H FDR	Log 2 FC (> 1.5 fold change	*P*‐value	B‐H FDR
let‐7b‐5p	—	0.23	0.0182	0.0428	—	—	—	—	—	—	—	—	—	—	—	—
miR‐100‐5p	—	0.46	0.003	0.0136	—	—	—	—	—	—	—	—	—	—	—	—
miR‐106b‐3p	—	0.43	0.0082	0.0224	—	—	—	—	—	—	—	—	—	—	—	—
miR‐10a‐5p	—	0.32	0.0372	0.0507	—	—	—	—	—	—	—	—	—	—	—	—
miR‐10b‐3p	—	0.35	0.0227	0.0442	—	—	—	—	—	—	—	—	—	—	—	—
miR‐10b‐5p	—	0.45	0.0093	0.0244	—	—	—	—	—	—	0.75	0.041	0.0501	—	—	—
miR‐122‐5p	0.66	0.66	0.0004	0.0091	0.95	0.0125	0.0395	—	—	—	—	—	—	0.86	0.0239	0.0451
miR‐1246‐5p	—	0.32	0.0416	0.0516	—	—	—	—	—	—	—	—	—	—	—	—
miR‐1249‐3p	—	—	—	—	—	—	—	‐0.62	0.0251	0.0552	—	—	—	—	—	—
miR‐125b‐1‐5p	—	0.3	0.0216	0.0446	—	—	—	—	—	—	—	—	—	—	—	—
miR‐127‐5p	—	0.51	0.0082	0.0233	1.02	0.0092	0.0776	—	—	—	—	—	—	0.82	0.0302	0.0399
miR‐1296‐5p	—	—	—	—	‐0.75	0.0111	0.0468	—	—	—	0.72	0.0228	0.0418	—	—	—
miR‐134‐5p	—	0.4	0.0495	0.0553	1.02	0.0156	0.0395	—	—	—	—	—	—	—	—	—
miR‐136‐5p	—	—	—	—	1.2	0.001	0.0253	—	—	—	—	—	—	0.76	0.04	0.0352
miR‐143‐3p	—	—	—	—	‐0.66	0.0251	0.0397	—	—	—	—	—	—	—	—	—
miR‐144‐3p	—	0.39	0.0231	0.0438	—	—	—	—	—	—	—	—	—	—	—	—
miR‐144‐5p	—	—	—	—	—	—	—	—	—	—	0.69	0.0212	0.0466	—	—	—
miR‐145‐5p	—	0.48	0.0065	0.0193	—	—	—	—	—	—	—	—	—	—	—	—
miR‐150‐5p	—	0.45	0.005	0.0179	—	—	—	—	—	—	—	—	—	—	—	—
miR‐155‐5p	—	—	—	—	—	—	—	—	—	—	0.84	0.0181	0.0498	—	—	—
miR‐17‐3p	—	0.44	0.0291	0.0496	—	—	—	—	—	—	—	—	—	—	—	—
miR‐181a‐1‐3p	—	0.39	0.0222	0.0445	—	—	—	—	—	—	—	—	—	—	—	—
miR‐183‐5p	—	—	—	—	—	—	—	—	—	—	0.93	0.0083	0.0457	—	—	—
miR‐190a‐5p	—	0.53	0.0005	0.0068	—	—	—	—	—	—	—	—	—	0.79	0.0062	0.0409
miR‐192‐5p	—	0.52	0.0008	0.0068	—	—	—	—	—	—	—	—	—	0.62	0.0404	0.0333
miR‐193a‐3p	—	0.39	0.0049	0.0186	0.66	0.0461	0.0507	—	—	—	—	—	—	0.61	0.0405	0.0314
miR‐193a‐5p	—	0.48	0.0028	0.0136	—	—	—	—	—	—	—	—	—	0.76	0.0231	0.0508
miR‐193b‐3p	—	0.52	0.002	0.0136	0.8	0.0095	0.0601	—	—	—	—	—	—	—	—	—
miR‐210‐3p	0.61	0.61	0.0026	0.0136	—	—	—	—	—	—	—	—	—	0.78	0.0274	0.0402
miR‐214‐3p	—	—	—	—	‐0.7	0.013	0.0365	—	—	—	0.88	0.0076	0.0836	—	—	—
miR‐215‐5p	—	0.33	0.0347	0.0526	—	—	—	—	—	—	—	—	—	—	—	—
miR‐23a‐5p	—	—	—	—	0.85	0.0318	0.0447	—	—	—	—	—	—	—	—	—
miR‐23b‐3p	—	0.27	0.0455	0.0544	—	—	—	—	—	—	—	—	—	—	—	—
miR‐24‐2‐5p	—	0.39	0.0395	0.0518	—	—	—	—	—	—	—	—	—	—	—	—
miR‐29a‐5p	—	0.45	0.0012	0.0091	—	—	—	—	—	—	—	—	—	—	—	—
miR‐29c‐5p	—	0.45	0.0105	0.0265	—	—	—	—	—	—	—	—	—	—	—	—
miR‐3200‐3p	0.61	0.61	0.0004	0.0068	—	—	—	—	—	—	—	—	—	—	—	—
miR‐320a‐3p	—	0.38	0.0254	0.0468	—	—	—	—	—	—	—	—	—	—	—	—
miR‐320b‐1‐3p	—	0.33	0.0284	0.0497	—	—	—	—	—	—	—	—	—	—	—	—
miR‐326‐3p	—	—	—	—	0.78	0.0409	0.0517	—	—	—	—	—	—	—	—	—
miR‐33b‐5p	—	—	—	—	1.02	0.0124	0.0448	—	—	—	—	—	—	—	—	—
miR‐34a‐5p	—	0.29	0.0385	0.0515	—	—	—	—	—	—	—	—	—	—	—	—
miR‐3615‐3p	—	0.31	0.019	0.0418	—	—	—	—	—	—	—	—	—	—	—	—
miR‐365a‐3p	—	—	—	—	—	—	—	—	—	—	0.8	0.0105	0.0385	—	—	—
miR‐370‐3p	—	0.35	0.0321	0.0509	0.78	0.0249	0.0420	—	—	—	—	—	—	—	—	—
miR‐375‐3p	—	0.31	0.0355	0.0515	—	—	—	—	—	—	—	—	—	—	—	—
miR‐376b‐3p	0.66	0.66	0.012	0.0292	1.42	0.0067	0.0848	—	—	—	—	—	—	—	—	—
miR‐377‐3p	—	—	—	—	1.08	0.023	0.0485	—	—	—	—	—	—	—	—	—
miR‐378a‐3p	0.62	0.62	0.0008	0.0078	0.78	0.0368	0.0490	—	—	—	—	—	—	0.86	0.0192	0.0634
miR‐4440‐3p	—	—	—	—	—	—	—	—	—	—	0.91	0.0315	0.0495	—	—	—
miR‐4508‐5p	—	—	—	—	0.79	0.0457	0.0526	—	—	—	—	—	—	—	—	—
miR‐451a‐3p	—	0.32	0.041	0.0518	—	—	—	—	—	—	—	—	—	—	—	—
miR‐4532‐5p	0.66	0.66	0.0033	0.0141	1.01	0.0226	0.0520	—	—	—	—	—	—	1.04	0.024	0.0396
miR‐483‐5p	0.59	0.59	0.002	0.0124	0.85	0.0249	0.0450	—	—	—	—	—	—	0.84	0.0324	0.0356
miR‐487b‐3p	—	0.4	0.0351	0.0520	—	—	—	—	—	—	—	—	—	—	—	—
miR‐490‐3p	—	—	—	—	—	—	—	—	—	—	—	—	—	0.73	0.05	0.0367
miR‐500a‐3p	—	0.3	0.0271	0.0486	—	—	—	—	—	—	—	—	—	—	—	—
miR‐501‐5p	—	0.51	0.0002	0.0068	—	—	—	—	—	—	—	—	—	0.63	0.0132	0.0381
miR‐505‐3p	—	0.46	0.0058	0.0198	—	—	—	—	—	—	—	—	—	—	—	—
miR‐548q‐5p	—	0.32	0.0197	0.0420	—	—	—	—	—	—	—	—	—	—	—	—
miR‐550a‐1‐3p	—	0.55	0.0005	0.0057	—	—	—	—	—	—	—	—	—	—	—	—
miR‐576‐3p	—	—	—	—	0.72	0.0239	0.0465	—	—	—	—	—	—	0.6	0.0359	0.0365
miR‐6087‐3p	—	0.51	0.0033	0.0132	—	—	—	—	—	—	—	—	—	—	—	—
miR‐6087‐5p	—	0.47	0.0059	0.0192	0.77	0.0317	0.0472	—	—	—	—	—	—	0.78	0.0212	0.0560
miR‐627‐5p	—	0.42	0.0335	0.0519	0.83	0.0426	0.0513	—	—	—	—	—	—	—	—	—
miR‐660‐3p	0.63	0.63	0.0001	0.0068	—	—	—	—	—	—	—	—	—	—	—	—
miR‐671‐5p	—	—	—	—	0.67	0.0101	0.0511	—	—	—	—	—	—	—	—	—
miR‐7‐1‐5p	—	0.34	0.0059	0.0183	—	—	—	—	—	—	—	—	—	—	—	—
miR‐7641‐1‐3p	—	0.37	0.019	0.0432	—	—	—	—	—	—	—	—	—	1.04	0.0005	0.0066
miR‐874‐3p	—	0.32	0.0441	0.0537	—	—	—	—	—	—	—	—	—	—	—	—
miR‐99a‐5p	—	0.50	0.0021	0.0119	—	—	—	—	—	—	—	—	—	—	—	—

B‐H FDR, Benjamini‐Hochberg False Discovery Rate; NPN, non‐progressors normal; NPO, non‐progressors obese; PN, progressors normal; PO, Progressors Obese.

### 3.3 Impact of BMI on disease progression associated circulating miRNA

**Figure 2:**
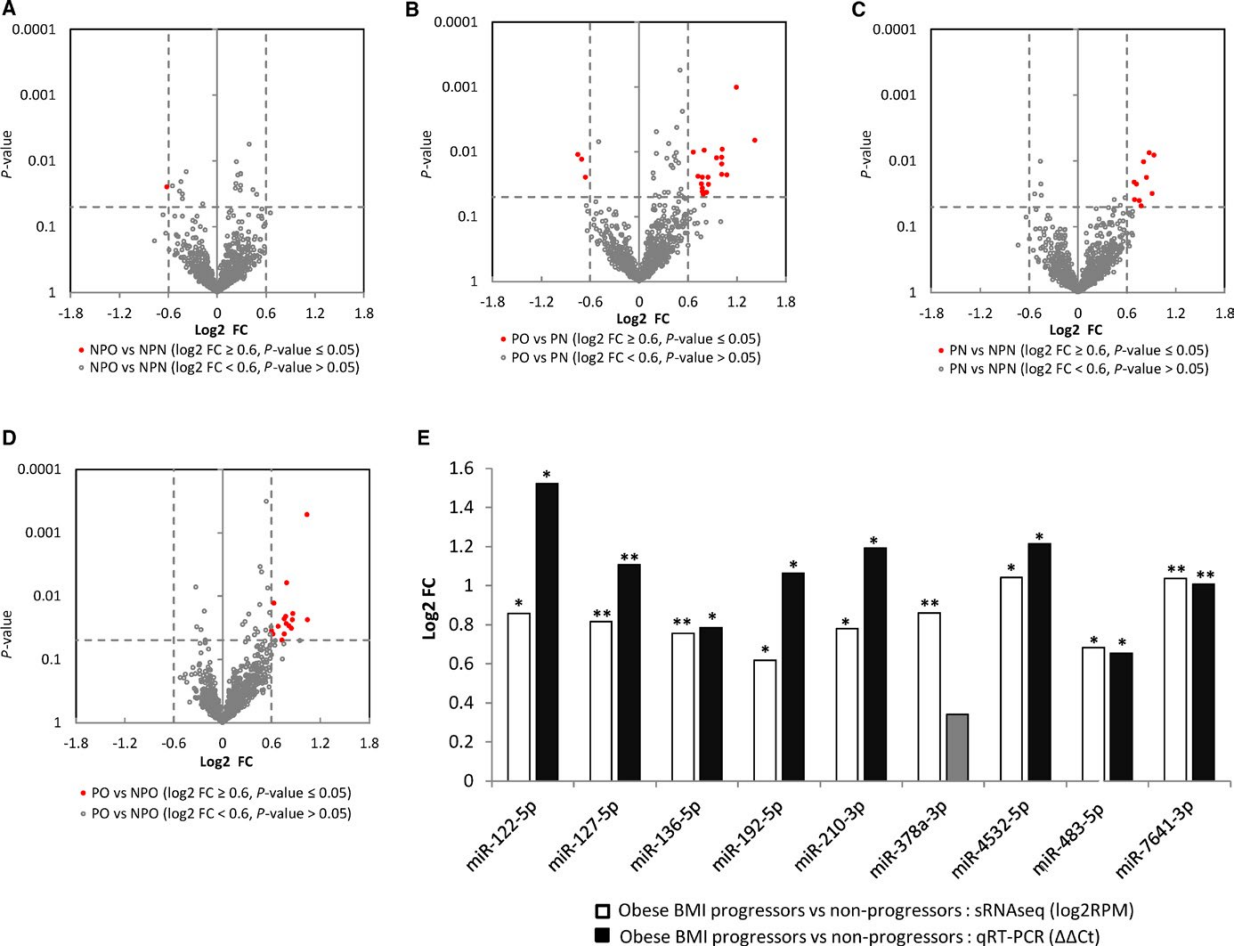
Impact of BMI on miRNA spectra in progressors and non‐progressors. A, Volcano plot of log2 fold change (log2FC) vs *P*‐value between obese non‐progressors (NPO) and normal non‐progressors (NPN), (B) obese progressors (PO) and normal progressors (PN), (C) normal progressors (PN) and normal non‐progressors (NPN), and (D) obese progressors (PO) and obese non‐progressors (NPO). Grey unfilled circles represent miRNAs with a *P* > 0.05, black circles represent miRNAs with a *P* < 0.05, and a Log2FC between ±0.28 and ±0.58 (Fold change between 1.2 and 1.5, respectively), and red circles represent miRNAs with a *P* < 0.05, and a log2FC>±0.58 (fold change greater than 1.5). E, qRT‐PCR results for 9 selected miRNAs (normalized to the average of miR‐16‐5p and miR‐21‐5p) between obese BMI progressors vs obese BMI non‐progressors (PO vs NPO). Values are represented as log2FC (Y‐axis) and the miRNA identity is indicated on the X‐axis. The qRT‐PCR data (black bars) is derived from ΔΔCt values and the sRNAseq data (white bars) is derived from log2RPM values.Single asterisks indicate a *P* < 0.05, double asterisks indicate a *P* < 0.01 Grayed out values without an asterisks indicate an insignificant result

We also looked at the impact of BMI on the profile of circulating miRNA between progressors and non‐progressors, as obesity is a well‐known risk factor for developing T2DM. Based on BMI, we grouped the individuals into 4 groups: Non‐progressors with normal BMI (BMI range 18‐24.9, n = 23) (NPN), obese non‐progressors (BMI range >30, n = 39) (NPO), progressors with normal BMI (BMI range 18‐24.9, n = 25) (PN), and obese progressors (BMI range >30, n = 41) (PO). We excluded individuals categorized as overweight (BMI from 24.9 to 29.9), so that we could observe a stronger effect of BMI on circulating miRNA. Among the non‐progressors, between normal and obese groups (NPO vs NPN), we identified a single miRNA (miR‐1249‐3p) showing lower concentration in the obese group (>1.5 fold concentration difference and a *P* < 0.05) (Figure 2A, Table 2). In contrast, in the progressor groups (PO vs PN), we identified 23 miRNAs, all except three (miR‐143‐3p, miR‐214‐3p and miR‐1296‐5p) of which were elevated in the obese (PO) group compared to normal (PN) (Figure 2B, Table 2). When comparing progressors and non‐progressors with normal BMI (PN vs NPN), we observed 10 differentially expressed miRNAs (Figure 2C, Table 2), whereas 17 miRNAs (Figure 2D, Table 2) showed concentration changes in obese individuals (PO vs NPO), of which 14 also showed significant changes between progressors and non‐progressors as a whole (Table 2). Of the 8 miRNAs we originally identified with significant concentration differences between progressors and non‐progressors, 6 of them (miR‐122‐5p, miR‐210‐3p, miR‐378a‐3p, miR‐4532‐5p, miR‐483‐5p, and miR‐7641‐3p) showed greater changes in the obese progressors vs non‐progressors comparison (PO vs NPO) (Figure 2E), suggesting that these T2DM progression‐associated circulating miRNAs are exacerbated by obesity. Our qRT‐PCR results confirmed the statistically significant concentration difference for miR‐122‐5p, miR‐210‐3p, miR‐4532‐5p, miR‐483‐5p, and miR‐7641‐3p, as wells as other miRNAs such as miR‐127‐5p, miR‐192‐5p, and miR‐136‐3p, between the obese progressor and obese non‐progressor groups (Figure 2E).

### 3.4 Novel miRNA and other types of extracellular RNA in circulation

**Figure 3:**
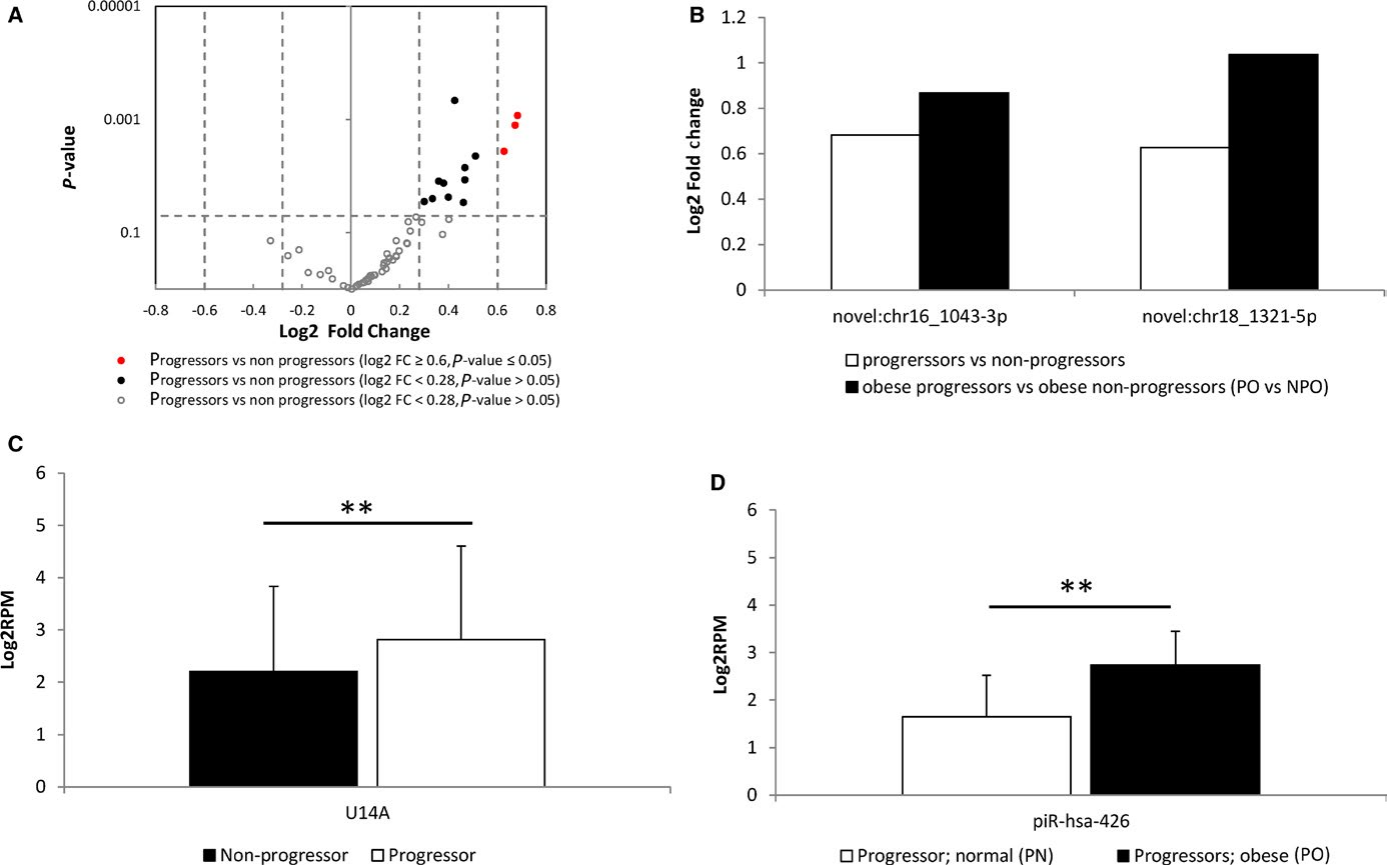
Novel miRNAs, and other small RNAs associated with T2DM progression._A, Volcano plot of log2 fold change (log2FC) vs p‐value between progressors and non‐progressors. Grey unfilled circles represent miRNAs with a *P* > 0.05, black circles represent miRNAs with a *P* < 0.05, and a Log2FC between ±0.28 and ±0.58 (Fold change between 1.2 and 1.5, respectively), and red circles represent miRNAs with a *P* < 0.05, and a log2FC>±0.58 (fold change greater than 1.5). B, Log2FC values for the 2 novel miRNAs showing significant concentration changes between all progressors and non‐progressors (white) and obese progressors and obese non‐progressors (grey) (see Supplemental Table S2). (C) Log2RPM values showing differences between non‐progressors (black) and progressors (white) for the snoRNA, U14A. (B) Log2RPM values showing differences between normal BMI progressors (PN, white), and obese progressors (PO, dotted) for the piRNA, piR‐426. A double asterisk indicates a *P* < 0.01

In addition to the known miRNAs, we identified 13 novel miRNA candidates that showed significant concentration changes between progressors and non‐progressors (*P* < 0.05), of which 3 also had a fold change >1.5 (Figure 3A: Red circles, Supplementary Table S3) and 10 had a fold change <1.5, but>1.2 (Figure 3A: Black circles, Supplementary Table S3). We also looked at the potential impact of BMI on the novel miRNA candidates and found that 9 were significantly elevated in the obese progressors vs obese non‐progressors comparison (PO vs NPO) (Supplementary Table S3). Of the 3 novel miRNAs we originally identified that showed significant fold changes >1.5 between progressors and non‐progressors, 2 were elevated in the PO vs NPO comparison (Figure 3B, Supplementary Table S3.) Besides miRNAs, our mapping pipeline also provides reads mapped to other types of RNA (Table 1). We identified a snoRNA, U14A, that was elevated in progressors compared to non‐progressors (log2 fold change = 0.61, *P* = 0.001493); and a piRNA, piRNA‐426, that is elevated in PO compared to PN (log2 fold change = 1.08, *P* = 6.94088E‐06) (Figure 3C,D).

### 3.5 Panels of circulating miRNAs to predict the progression of prediabetes to T2DM

**Figure 4:**
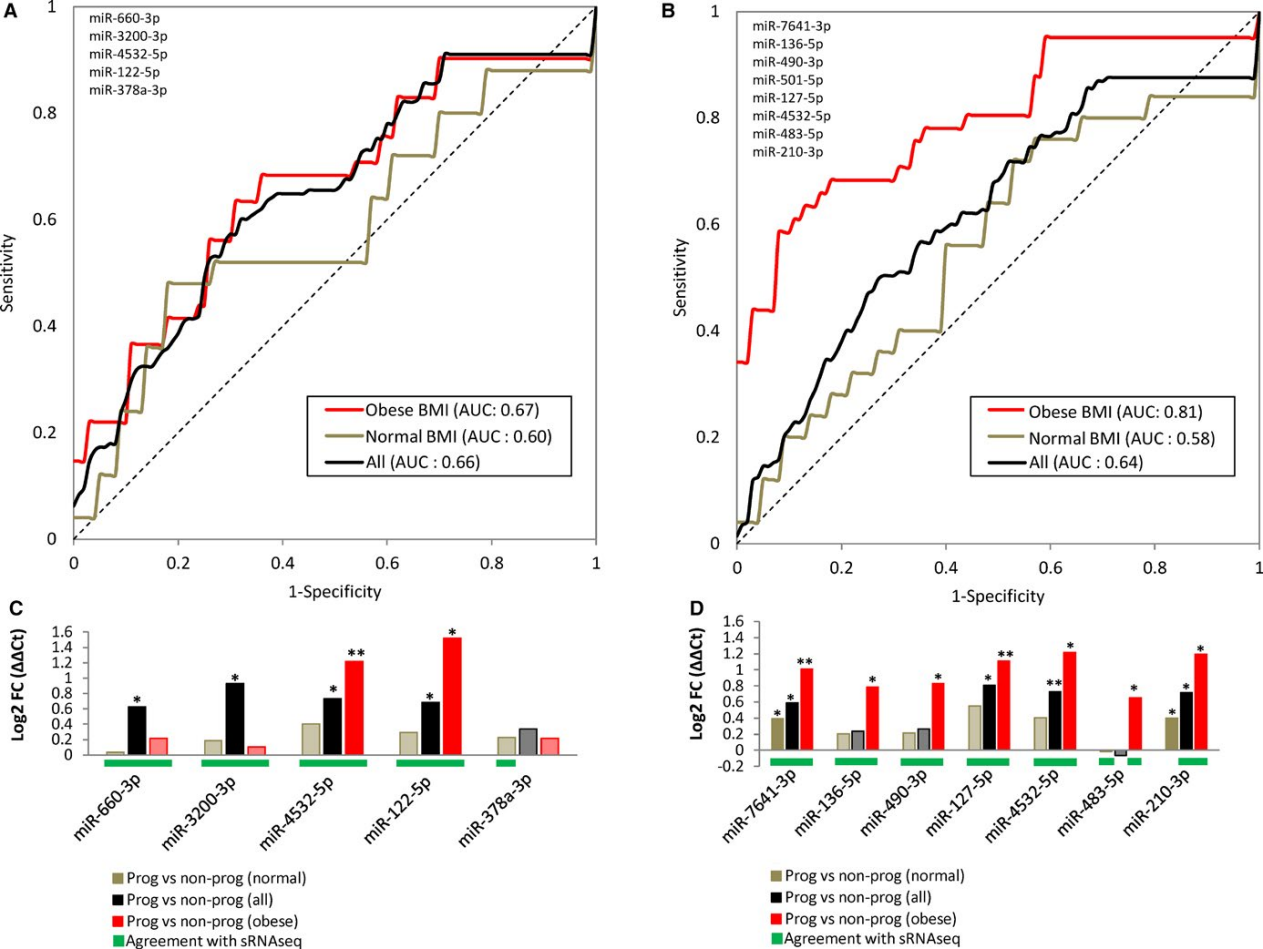
ROC analysis of miRNA biomarker panels. Receiver Operating Characteristic (ROC) analysis for predictive miRNA panels for all samples between progressors and non‐progressors (A) and obese progressors and obese non‐porgressors (B). Sensitivity and specificity are plotted against each other, and the average Area Under the Curve (AUC) is given for each patient group tested. All patients = black, obese patients = red, normal BMI patients = brown. C, qRT‐PCR results for the five‐miRNA panel (normalized to the average of miR‐16‐5p and miR‐21‐5p) between all progressors and non‐progressors (black), obese BMI progressors and non‐progressors (red), and normal BMI progressors and non‐progressors (brown). D, qRT‐PCR results for the eight‐miRNA panel (normalized to the average of miR‐16‐5p and miR‐21‐5p) between all progressors and non‐progressors (black), obese BMI progressors and non‐progressors (red), and normal BMI progressors and non‐progressors (brown). Values are represented as log2FC (ΔΔCt) on the Y‐axis and the miRNA identity is indicated on the X‐axis. Single asterisks indicate a *P* < 0.05, double asterisks indicate a *P* < 0.01. Grayed out values without an asterisks indicate an insignificant result. Green bars below values indicate agreement with sRNAseq (see Table 2)

We identified a five‐miRNA panel (miR‐660‐3p, miR‐3200‐3p, miR‐4532‐5p, miR‐122‐5p, and miR‐378a‐3p) that was able to discriminate progressors from non‐progressors using receiver operating characteristic (ROC) analysis (AUC = 0.66) (Figure 4A). As expected, for the five‐miRNA panel the AUC was slightly higher among samples from obese individuals (AUC = 0.67) compared to non‐obese individuals (AUC = 0.60) (Figure 4A). This prompted the identification of an improved predictive panel for obese patients since BMI is one of the key predictive factors for the development of T2DM. A panel of eight miRNAs (miR‐7641‐3p, miR‐136‐5p, miR‐490‐3p, miR‐501‐5p, miR‐127‐5p, miR‐4532‐5p, miR‐483‐5p, miR‐210‐3p) showed a good separation between obese progressors and obese non‐progressors with an AUC of 0.81 (Figure 4B). The miRNA panels performed better for their predictive value when compare to the threshold FPG alone (Supplemental Figure S3). We did qRT‐PCR validation of the miRNAs in these panels and found that they agreed well with the sRNAseq results (Figure 4C, D).

### 3.6 Downstream pathways and biological processes associated with dysregulated miRNAs

**Figure 5:**
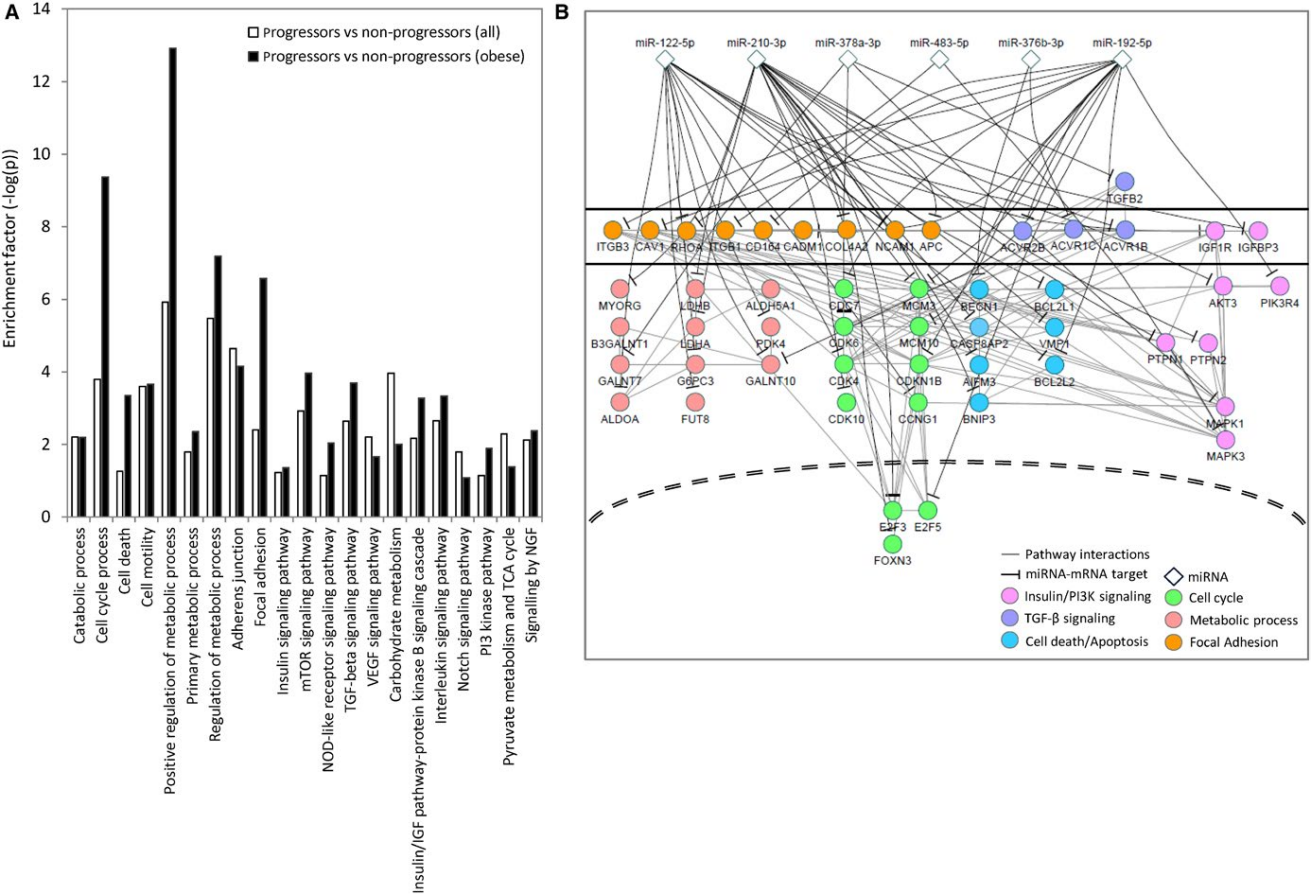
Biological pathways and processes that might be affected by the dysregulated miRNAs. A, Enrichment of different biological processes and pathways regulated by miRNAs associated with T2DM progression in all patients (white bars) and obese patients (black bars). The miRTar database (*mirtarbase.mbc.nctu.edu.tw/*) was used to identify validated miRNA targets for gene enrichment (validated by at least two different techniques) and gene enrichment analysis was performed with DAVID (Database for Annotation, Visualization and Integrated Discovery, https://david.ncifcrf.gov/). B, Details of the gene targets (mRNAs) of some of the pathways and processes associated with T2DM progression. MiRNAs (diamonds) associated with T2DM progression are at the top. Different target mRNAs associated with different pathways and processes are shown (Pink—Insulin/PI3K, Purple—TGF‐β, blue—cell death/apoptosis, green—cell cycle, red—metabolic process, orange—focal adhesion) with black lines depicting miRNA‐mRNA interactions, and light grey lines depicting protein‐protein interactions between pathway components as determined by STRING database (https://string-db.org/)

Using validated miRNA targets, we explored the possible biological processes/pathways that might be affected by the dysregulated miRNAs (Figure 5). A number of signal transduction related processes including the insulin signaling pathway, mTOR signaling pathway, TGF‐β signaling pathway, and VEGF signaling pathway, were enriched in this analysis (Figure 5A). In addition, there was enrichment for other processes including cell cycle, cell death, metabolism, and focal adhesion (Figure 5A). Many of the direct gene targets in these pathways and processes represent a diverse set of family members, including ligands, receptors, intracellular mediators, and downstream effectors, with miRNAs targeting multiple components of the same pathway at different points (Figure 5B, Table 3).

**Table 3:** Selected miRNAs targets from biological processes and pathways associated with T2DM progression

miRNA	Target	Process/pathway	Description
miR‐122‐5p	CCNG1	Cell cycle	Cyclin G1
miR‐192‐5p	CDC7	Cell cycle	Cell division related kinase
miR‐210‐3p	CDK10	Cell cycle	Cyclin Dependent Kinase
miR‐122‐5p	CDK4	Cell cycle	Cyclin Dependent Kinase
miR‐378a‐3p	CDK6	Cell cycle	Cyclin Dependent Kinase
miR‐192‐5p	CDKN1B	Cell cycle	Cyclin Dependent Kinase inhibitor
miR‐210‐3p	E2F3	Cell cycle	transcription factor
miR‐192‐5p	E2F5	Cell cycle	transcription factor
miR‐210‐3p	FOXN3	Cell cycle	transcription factor
miR‐192‐5p	MCM10	Cell cycle	DNA helicase
miR‐192‐5p	MCM3	Cell cycle	DNA helicase
miR‐210‐3p	MCM3	Cell cycle	DNA helicase
miR‐210‐3p	AIFM3	Cell death/Apoptosis	Apoptosis‐inducing factor
miR‐122‐5p	BAX	Cell death/Apoptosis	BCL2 family
miR‐192‐5p	BCL2	Cell death/Apoptosis	BCL2 family
miR‐122‐5p	BCL2L1	Cell death/Apoptosis	BCL2 family
miR‐122‐5p	BCL2L2	Cell death/Apoptosis	BCL2 family
miR‐376b‐3p	BECN1	Cell death/Apoptosis	BCL2 interactor
miR‐210‐3p	BNIP3	Cell death/Apoptosis	BCL2 family
miR‐210‐3p	CASP8AP2	Cell death/Apoptosis	Caspase complex
miR‐210‐3p	VMP1	Cell death/Apoptosis	BCL2 interactor
miR‐210‐3p	APC	Focal Adhesion	Cell‐cell adhesion
miR‐192‐5p	CADM1	Focal Adhesion	Cell‐cell adhesion
miR‐192‐5p	CAV1	Focal Adhesion	Integrin pathway
miR‐192‐5p	CD164	Focal Adhesion	Cell‐cell adhesion
miR‐210‐3p	COL4A2	Focal Adhesion	Cell‐cell adhesion
miR‐192‐5p	ITGB1	Focal Adhesion	Integrin pathway
miR‐192‐5p	ITGB3	Focal Adhesion	Integrin pathway
miR‐122‐5p	NCAM1	Focal Adhesion	Cell‐cell adhesion
miR‐210‐3p	NCAM1	Focal Adhesion	Cell‐cell adhesion
miR‐122‐5p	RHOA	Focal Adhesion	Rho/ROCK pathway
miR‐483‐5p	RHOA	Focal Adhesion	Rho/ROCK pathway
miR‐122‐5p	AKT3	Insulin/PI3K signaling	Downstream mediator
miR‐122‐5p	IGF1R	Insulin/PI3K signaling	Receptor
miR‐210‐3p	IGFBP3	Insulin/PI3K signaling	IGF‐associated
miR‐378a‐3p	MAPK1	Insulin/PI3K signaling	Downstream mediator
miR‐483‐5p	MAPK3	Insulin/PI3K signaling	Downstream mediator
miR‐192‐5p	PIK3R4	Insulin/PI3K signaling	Downstream mediator
miR‐210‐3p	PTPN1	Insulin/PI3K signaling	Negative regulator
miR‐210‐3p	PTPN2	Insulin/PI3K signaling	Negative regulator
miR‐210‐3p	ALDH5A1	Metabolic process	Aldehyde Dehydrogenase
miR‐122‐5p	ALDOA	Metabolic process	Fructose‐bisphosphate aldolase
miR‐192‐5p	B3GALNT1	Metabolic process	Beta‐1,3‐N‐acetylgalactosaminyltransferase
miR‐122‐5p	FUT8	Metabolic process	Fucosyltransferase 8
miR‐122‐5p	G6PC3	Metabolic process	Glucose‐6‐Phosphatase Catalytic Subunit
miR‐122‐5p	GALNT10	Metabolic process	N‐Acetylgalactosaminyltransferase
miR‐378a‐3p	GALNT7	Metabolic process	N‐Acetylgalactosaminyltransferase
miR‐210‐3p	LDHA	Metabolic process	Lactate dehydrogenase
miR‐210‐3p	LDHB	Metabolic process	Lactate dehydrogenase
miR‐210‐3p	MYORG	Metabolic process	Glycosidase
miR‐210‐3p	ACVR1B	TGF‐β signaling	Receptor
miR‐376b‐3p	ACVR1C	TGF‐β signaling	Receptor
miR‐192‐5p	ACVR2B	TGF‐β signaling	Receptor
miR‐378a‐3p	TGFB2	TGF‐β signaling	Ligand

## 4 DISCUSSION

Currently there is no tool that can accurately predict the development of T2DM from prediabetes. By comparing the plasma circulating RNA profiles between two prediabetic groups—one that progresses to T2DM after 5 years and the other group remaining at the prediabetic stage—we identified 57 miRNAs showing significant concentration differences between the two groups at baseline (prediabetic stage). When limiting the comparison to obese progressors vs obese non‐progressors most of these miRNAs showed even greater concentration differences. We validated some of those miRNAs by qRT‐PCR, suggesting that the improvements in library construction and data analysis translate to better cross‐platform data consistency, as we have demonstrated previously.^14^, ^15^ Our sequence analysis pipeline also provides novel miRNA candidates and mapping results for other types of RNAs (such as snoRNA and piRNA) so that we can investigate the possible association of these RNAs with T2DM progression or obesity. Even with these improvements in library construction and data analysis, there are still limitations to profiling miRNAs in a large cohort of patients. Because of the lengthy small RNA library preparation process and complicated data analysis to extract useful information, the NGS based small RNA analysis does not suitable for routine measurement of patient plasma miRNAs in the clinic. Rather, the current sRNAseq method is ideal for discovery‐based studies, and methods like qRT‐PCR is are more appropriate for validation and clinic applications.

While other groups have examined the miRNA spectra between individuals with T2DM and healthy controls, few studies have looked at miRNAs that may associate with the progression from prediabetes to T2DM. The samples from the METSIM study are unique in that the study focused on global molecular comparisons, from microbiome to genome, between progressors and non‐progressors, to identify predictive biomarkers for the manifestation of T2DM. Not surprisingly, the concentration changes of specific circulating RNA species that we observed at the prediabetic stage was not as high as what has been observed between T2DM and non‐T2DM healthy individuals, where the difference in disease burden is more pronounced. Similarly, previous studies that have looked at prediabetes and T2DM have used miRNAs for diagnostic purpose and primarily done comparisons among NGT, prediabetes, and T2DM patient cohorts but not on the difference between progressors and non‐progressors of prediabetic patients at baseline.^21^, ^22^, ^23^

Of the 57 miRNAs showing concentration differences between the progressor and non‐progressor groups, 26 of them (miR‐10b‐5p, miR‐144‐3p, miR‐451a‐5p, miR‐192‐5p, miR‐125b‐5p, miR‐101‐5p, miR‐122‐5p, miR‐150‐5p, miR‐17‐3p, miR‐193a‐5p, miR‐193b‐3p, miR‐29c‐5p, miR‐320a‐3p, miR‐320b‐3p, miR‐550a‐3p, miR‐100‐5p, miR‐10a‐5p, miR‐181a‐3p, miR‐190a‐5p, miR‐136‐5p, miR‐375‐3p, miR‐487b‐3p, miR‐7‐5p, miR‐210‐3p, miR‐378a‐3p, and miR‐99a‐5p) have been previously described as being associated with T2DM based on our recent meta‐analysis of the field.^10^ We selected 14 miRNAs for qRT‐PCR validation and were able to confirm the concentration changes for 10 of these miRNAs (miR‐122‐5p, miR‐127‐5p, miR‐192‐5p, miR‐210‐3p, miR‐3200‐3p, miR‐375‐3p, miR‐376b‐3p, miR‐4532‐5p, and miR‐660‐3p, and miR‐7641‐3p). Some miRNAs could not be verified with qRT‐PCR, probably due to the specific qPCR platform used and high‐false negative rate associated with qRT‐PCR as previously reported.^24^, ^25^, ^26^

We investigated the impact of obesity on circulating miRNA and found a notable effect among progressors (Table 2). Conversely, in the non‐progressors the impact of obesity was minimal (only one affected miRNA observed). As expected, obesity also affects miRNAs associated with prediabetic–to‐T2DM transition (16 miRNAs showed concentration changes between obese progressors and non‐progressors). Of the 16 obesity‐associated miRNAs linked with the transition to T2DM, we followed up with nine selected miRNAs for qRT‐PCR validation, and confirmed concentration changes of miR‐122‐5p, miR‐127‐5p, miR‐136‐3p, miR‐192‐5p, miR‐210‐3p, miR‐4532‐5p, miR‐483‐5p, and miR‐7641‐3p. As mentioned above, these miRNAs have been previously implicated in T2DM. MiR‐122‐5p is a liver‐enriched miRNA that acts in response to insulin to control lipid metabolism.^27^, ^28^ Elevated miR‐122‐5p levels in serum or plasma have been previously reported to be associated with metabolic syndrome, T2DM, insulin resistance, and obesity.^29^, ^30^, ^31^ MiR‐192 is a liver and pancreatic‐enriched miRNA that has been shown to be associated with insulin resistance and is elevated in the plasma of prediabetic individuals, suggesting that elevated levels of miR‐192‐5p in circulation may signal progression towards T2DM.^31^, ^32^ MiR‐210‐3p has been previously shown to be involved in adipogenesis, is differentially expressed in visceral adipose tissues in obese T2DM individuals, and is associated with gestational diabetes in obese women.^33^, ^34^, ^35^, ^36^

Using validated miRNA targets, we explored the possible biological processes/pathways that might be affected by the dysregulated miRNAs. A number of signal transduction related processes that play important roles in T2DM pathophysiology, including the insulin signaling pathway, mTOR signaling pathway, TGF‐β signaling pathway, and VEGF signaling pathway^37^, ^38^, ^39^, ^40^ were enriched in this analysis. In addition, these dysregulated miRNAs showed enrichment for other processes including cell cycle, cell death, metabolism, and focal adhesion, which are also involved in T2DM disease progression^41^, ^42^, ^43^

. Interestingly, many of these pathways and processes showed stronger enrichment in the obese progressors vs non‐progressors, compared to progressors vs non‐progressors as a whole (Figure 5A). Furthermore, the direct gene targets represent a diverse set of family members associated with these pathways and processes (see Figure 5B, Table 3). For example in the cell cycle, miRNAs we find to be associated with T2DM progression directly regulate the expression of cyclins (CCNG1), cyclins‐dependent kinases (CDK10, CDK4, CDK6), DNA helicases (MCM10, MCM3), and transcription factors (E2F3, E2F5, FOXN3). In insulin/insulin‐like signaling (which acts through components of the PI3K pathway) the receptor (IGF1R), as well as downstream mediators (AKT3, PIK3R4, MAPK1, MAPK3) and external regulators (PTPN1, PTPN2) are targeted by these miRNAs. In the case of the TGF‐ β pathway, the miRNAs seem to be strictly targeting upstream components, such as ligands (TGFB2) and receptors (ACVR1B, ACVR1C, ACVR2B. Targeted genes associated with metabolism/metabolic processes represented a wide‐array of enzyme associated with metabolic pathways. Some of these genes are associated with glycolysis/gluconeogenesis (ALDOA, G6PC3, MYORG) and glycan biosynthesis (B3GALNT1, GALNT7, GALNT10). Interestingly, some of these genes, such as ALDOA and PTPN1, have been to link obesity to T2DM.^44^, ^45^, ^46^, ^47^ We also identified two miRNA panels that showed moderate discriminatory ability to distinguish progressors from non‐progressors. As expected, these panels performed better when tested among obese individuals, rather than all individuals, or patients with a normal BMI, which performed the poorest. Interestingly, the best performance (AUC = 0.81) was achieved with the eight miRNA panel testing obese patients, which are at higher risk for developing T2DM. This is probably due to the panel contains several miRNAs (miR‐136‐5p, miR‐490‐3p, miR‐483‐5p) that show concentration differences between obese patients, but not when comparing all patients or patients with normal BMI. The results from qRT‐PCR of these miRNAs in obese and normal BMI patients as well as all patients agree with the findings (Figure 3B). We propose that the eight‐miRNA panel will have better clinical utility when testing obese prediabetic patients. The value of using a “discovery‐based” approach over a conventional candidate‐based approach is that new miRNAs not previously reported to be associated with a given disease or health condition may be identified. While many of the miRNAs we observed have been seen in other T2DM studies, the majority have no known association with T2DM or T2DM progression. We have also identified novel miRNAs not previously characterized, that are associated with T2DM progression and affected by obesity. With further validation, these miRNAs could be considered as promising predictive biomarkers for identifying prediabetic individuals that will progress to T2DM.

## CONFLICT OF INTERESTS

The authors declare no conflicts of interests.
