# Supplementary material for: Circulating RNAs as predictive markers for the progression of type 2 diabetes
Source: J Cell Mol Med. 2019 Feb 7;23(4):2753–68. doi: 10.1111/jcmm.14182 (PMC6433655; doi:10.1111/jcmm.14182)
Supplement: Supplementary file 1 [file JCMM-23-2753-s001.docx]

**Supplemental Table 1.** Patient information and small RNA sequencing read count information.

| **Patient #** | **GROUP** | **BMI** | **Fasting plasma glucose** | **Raw read count** | **Processed read** | **Read mapped to human** |
| --- | --- | --- | --- | --- | --- | --- |
| 1 | non-progressor | 31.1 | 6 | 8,057,647 | 3,312,293 | 1,609,718 |
| 2 | non-progressor | 24.1 | 5.9 | 18,953,603 | 2,010,784 | 527,602 |
| 3 | non-progressor | 26.9 | 6.3 | 6,837,176 | 3,573,606 | 1,739,745 |
| 4 | non-progressor | 26.3 | 5.8 | 6,082,275 | 3,152,795 | 1,631,136 |
| 5 | non-progressor | 29.6 | 5.9 | 13,387,411 | 1,415,582 | 448,911 |
| 6 | non-progressor | 31 | 6.1 | 19,861,223 | 9,181,953 | 2,273,607 |
| 7 | non-progressor | 32.2 | 6.1 | 6,616,842 | 3,689,731 | 2,051,264 |
| 8 | non-progressor | 23.7 | 6.3 | 6,760,358 | 2,848,454 | 1,182,769 |
| 9 | non-progressor | 26 | 6 | 7,424,830 | 4,977,805 | 2,668,482 |
| 10 | non-progressor | 22.9 | 5.6 | 12,898,314 | 9,615,226 | 4,418,385 |
| 11 | non-progressor | 30.5 | 6.2 | 19,194,084 | 3,953,470 | 1,383,613 |
| 12 | non-progressor | 27.4 | 5.9 | 8,719,286 | 422,664 | 84,919 |
| 13 | non-progressor | 38.1 | 5.9 | 24,280,995 | 8,475,743 | 3,111,520 |
| 14 | non-progressor | 28.2 | 5.9 | 5,230,012 | 2,092,851 | 939,056 |
| 15 | non-progressor | 25 | 6.3 | 7,848,789 | 3,117,328 | 1,274,948 |
| 16 | non-progressor | 27.8 | 5.9 | 19,175,547 | 3,003,305 | 927,443 |
| 17 | non-progressor | 27.3 | 6.1 | 6,359,898 | 2,625,796 | 1,182,460 |
| 18 | non-progressor | 24.2 | 5.9 | 8,489,856 | 4,199,803 | 1,590,233 |
| 19 | non-progressor | 34.1 | 6.9 | 10,248,375 | 8,304,103 | 3,103,041 |
| 20 | non-progressor | 31.8 | 6.1 | 4,768,673 | 2,590,189 | 944,416 |
| 21 | non-progressor | 31.4 | 6.2 | 22,433,277 | 2,170,983 | 434,412 |
| 22 | non-progressor | 28.9 | 6.1 | 13,046,746 | 3,054,588 | 1,372,057 |
| 23 | non-progressor | 26.7 | 6.7 | 19,488,674 | 1,533,779 | 452,454 |
| 24 | non-progressor | 32.4 | 6.5 | 6,428,218 | 4,271,364 | 2,630,238 |
| 25 | non-progressor | 27.8 | 6.4 | 8,568,554 | 3,558,253 | 1,658,614 |
| 26 | non-progressor | 32.2 | 5.8 | 9,774,241 | 6,581,046 | 3,095,150 |
| 27 | non-progressor | 27.2 | 6.2 | 12,714,420 | 1,205,680 | 302,959 |
| 28 | non-progressor | 30.3 | 6.3 | 8,737,133 | 6,276,159 | 2,712,341 |
| 29 | non-progressor | 27.2 | 6.3 | 9,907,805 | 5,422,360 | 2,959,050 |
| 30 | non-progressor | 28.5 | 6.1 | 9,151,841 | 4,696,291 | 2,513,902 |
| 31 | non-progressor | 34.2 | 5.7 | 5,671,240 | 4,092,427 | 2,392,278 |
| 32 | non-progressor | 28.2 | 5.7 | 7,562,811 | 1,970,381 | 849,414 |
| 33 | non-progressor | 26.6 | 6.2 | 4,991,786 | 2,167,568 | 929,617 |
| 34 | non-progressor | 25.8 | 6.1 | 7,359,813 | 2,929,483 | 1,375,290 |
| 35 | non-progressor | 29.2 | 6.1 | 9,606,726 | 3,248,334 | 1,467,940 |
| 36 | non-progressor | 29.8 | 5.6 | 15,763,697 | 9,010,318 | 3,368,328 |
| 37 | non-progressor | 28.9 | 5.8 | 12,753,627 | 4,188,030 | 2,184,195 |
| 38 | non-progressor | 30.8 | 6.7 | 9,376,923 | 7,695,582 | 4,878,768 |
| 39 | non-progressor | 28.2 | 6 | 13,090,717 | 5,076,432 | 2,702,993 |
| 40 | non-progressor | 26.8 | 6.4 | 8,579,927 | 6,532,318 | 3,950,439 |
| 41 | non-progressor | 27.1 | 6.5 | 11,013,043 | 5,621,333 | 2,800,554 |
| 42 | non-progressor | 21.8 | 6.3 | 18,720,586 | 13,418,421 | 8,859,690 |
| 43 | non-progressor | 27.6 | 5.8 | 6,275,112 | 5,402,725 | 3,361,277 |
| 44 | non-progressor | 27.6 | 6 | 7,083,662 | 2,779,167 | 1,036,918 |
| 45 | non-progressor | 28.2 | 5.7 | 12,411,744 | 6,582,330 | 2,612,684 |
| 46 | non-progressor | 30 | 6.3 | 24,215,460 | 1,976,909 | 654,654 |
| 47 | non-progressor | 25.8 | 6.4 | 9,367,973 | 6,053,175 | 3,570,324 |
| 48 | non-progressor | 30.6 | 5.7 | 8,700,292 | 6,313,419 | 3,216,216 |
| 49 | non-progressor | 27.7 | 6 | 6,801,876 | 4,776,553 | 2,630,569 |
| 50 | non-progressor | 28.9 | 6.6 | 7,827,594 | 6,013,617 | 1,740,671 |
| 51 | non-progressor | 22.8 | 5.9 | 8,165,387 | 5,898,498 | 3,683,777 |
| 52 | non-progressor | 31 | 6.1 | 18,559,270 | 2,808,833 | 587,238 |
| 53 | non-progressor | 24.3 | 6 | 11,406,065 | 5,856,936 | 2,613,453 |
| 54 | non-progressor | 33.4 | 6.9 | 20,163,500 | 2,517,724 | 627,560 |
| 55 | non-progressor | 29.4 | 6.5 | 13,661,843 | 2,464,171 | 483,334 |
| 56 | non-progressor | 26.8 | 6.4 | 9,997,448 | 5,593,517 | 2,906,074 |
| 57 | non-progressor | 29 | 5.7 | 15,158,722 | 1,914,098 | 741,218 |
| 58 | non-progressor | 22.3 | 6.1 | 13,283,993 | 7,098,964 | 2,990,424 |
| 59 | non-progressor | 33.8 | 6.1 | 14,184,526 | 4,180,875 | 865,818 |
| 60 | non-progressor | 24.8 | 6 | 36,895,705 | 30,256,845 | 18,553,865 |
| 61 | non-progressor | 30.7 | 6.2 | 14,280,320 | 6,803,610 | 2,545,854 |
| 62 | non-progressor | 29.2 | 6.7 | 12,797,505 | 7,085,021 | 2,731,864 |
| 63 | non-progressor | 33.3 | 6.2 | 11,464,268 | 9,821,689 | 4,832,382 |
| 64 | non-progressor | 29.8 | 5.8 | 7,466,016 | 1,748,882 | 522,272 |
| 65 | non-progressor | 29.6 | 5.6 | 12,253,635 | 10,115,070 | 5,492,845 |
| 66 | non-progressor | 27.2 | 5.8 | 18,098,215 | 1,470,191 | 449,549 |
| 67 | non-progressor | 26.5 | 5.8 | 6,105,776 | 4,087,480 | 1,987,466 |
| 68 | non-progressor | 26.6 | 6.3 | 10,769,978 | 6,815,903 | 3,403,025 |
| 69 | non-progressor | 33.4 | 6.1 | 14,553,487 | 12,656,505 | 8,199,746 |
| 70 | non-progressor | 27.9 | 5.9 | 24,502,745 | 2,142,914 | 410,313 |
| 71 | non-progressor | 26.4 | 6 | 15,291,377 | 8,979,444 | 3,687,696 |
| 72 | non-progressor | 29.1 | 5.7 | 9,692,130 | 7,773,276 | 3,065,250 |
| 73 | non-progressor | 28.1 | 6.5 | 10,927,654 | 7,626,402 | 3,201,678 |
| 74 | non-progressor | 32.1 | 5.7 | 9,524,790 | 4,777,980 | 1,695,643 |
| 75 | non-progressor | 30.2 | 6.2 | 15,955,013 | 10,339,758 | 5,005,269 |
| 76 | non-progressor | 27 | 5.9 | 17,042,914 | 9,308,231 | 2,355,630 |
| 77 | non-progressor | 24.5 | 6.4 | 10,731,214 | 7,652,163 | 1,523,784 |
| 78 | non-progressor | 33.3 | 6.6 | 7,230,387 | 5,022,582 | 2,287,437 |
| 79 | non-progressor | 25 | 6.4 | 10,048,052 | 8,114,176 | 4,721,166 |
| 80 | non-progressor | 31.2 | 6.3 | 9,412,923 | 5,508,586 | 2,628,135 |
| 81 | non-progressor | 29.6 | 6.2 | 8,382,132 | 6,109,130 | 3,595,367 |
| 82 | non-progressor | 23.3 | 5.7 | 7,889,216 | 5,154,318 | 1,430,997 |
| 83 | non-progressor | 23 | 6 | 7,646,577 | 5,747,889 | 1,790,711 |
| 84 | non-progressor | 31.8 | 6.2 | 12,624,856 | 11,279,146 | 6,732,917 |
| 85 | non-progressor | 35.1 | 5.8 | 10,494,105 | 7,488,290 | 3,508,291 |
| 86 | non-progressor | 25.5 | 6.6 | 24,862,437 | 1,930,656 | 397,805 |
| 87 | non-progressor | 23.7 | 6.3 | 11,353,559 | 6,678,256 | 2,863,813 |
| 88 | non-progressor | 35.5 | 6.1 | 4,581,083 | 1,996,812 | 751,113 |
| 89 | non-progressor | 26.4 | 6.4 | 12,192,722 | 7,894,491 | 3,434,780 |
| 90 | non-progressor | 29 | 5.9 | 4,602,717 | 3,715,885 | 2,012,905 |
| 91 | non-progressor | 27.1 | 5.7 | 14,479,237 | 9,739,279 | 4,203,350 |
| 92 | non-progressor | 25 | 5.7 | 13,053,926 | 11,206,186 | 5,024,032 |
| 93 | non-progressor | 26.5 | 6.2 | 8,975,881 | 5,466,606 | 2,887,584 |
| 94 | non-progressor | 24.8 | 5.6 | 11,566,804 | 7,568,275 | 3,635,176 |
| 95 | non-progressor | 28.4 | 5.8 | 12,153,596 | 4,017,274 | 920,808 |
| 96 | non-progressor | 20.5 | 6 | 7,956,099 | 4,639,485 | 2,282,092 |
| 97 | non-progressor | 32.9 | 5.6 | 10,411,502 | 2,917,118 | 1,556,765 |
| 98 | non-progressor | 32.2 | 5.9 | 10,048,773 | 8,449,906 | 4,966,649 |
| 99 | non-progressor | 27.9 | 5.7 | 4,615,620 | 2,682,261 | 847,941 |
| 100 | non-progressor | 33.1 | 5.9 | 7,373,826 | 4,133,677 | 1,527,193 |
| 101 | non-progressor | 26.4 | 6.1 | 12,900,921 | 4,071,149 | 851,606 |
| 102 | non-progressor | 25.5 | 6.3 | 9,955,492 | 6,429,127 | 1,669,448 |
| 103 | non-progressor | 26.8 | 6.4 | 14,902,687 | 2,863,990 | 494,219 |
| 104 | non-progressor | 29.4 | 5.9 | 15,096,142 | 5,287,020 | 840,838 |
| 105 | non-progressor | 28.1 | 6.5 | 15,785,632 | 2,933,428 | 529,312 |
| 106 | non-progressor | 25.2 | 6.1 | 13,282,718 | 4,620,934 | 686,406 |
| 107 | non-progressor | 27.9 | 5.7 | 26,328,468 | 2,002,298 | 228,740 |
| 108 | non-progressor | 30.4 | 5.8 | 16,198,843 | 5,430,987 | 1,723,880 |
| 109 | non-progressor | 28.7 | 6 | 17,769,546 | 3,235,682 | 661,025 |
| 110 | non-progressor | 31.5 | 5.7 | 10,129,732 | 5,525,212 | 1,313,137 |
| 111 | non-progressor | 29 | 5.6 | 8,392,211 | 4,487,060 | 1,626,005 |
| 112 | non-progressor | 29.6 | 6 | 22,061,008 | 10,641,824 | 1,353,589 |
| 113 | non-progressor | 36 | 6.6 | 31,747,753 | 20,354,631 | 2,599,871 |
| 114 | non-progressor | 33.4 | 6.3 | 15,957,084 | 7,074,887 | 1,601,033 |
| 115 | non-progressor | 27.6 | 6.8 | 7,188,278 | 4,198,801 | 1,511,102 |
| 116 | non-progressor | 23.5 | 5.7 | 5,658,785 | 2,550,843 | 805,478 |
| 117 | non-progressor | 28.4 | 5.8 | 13,412,376 | 2,637,336 | 392,755 |
| 118 | non-progressor | 32.9 | 6 | 8,947,176 | 2,119,493 | 469,246 |
| 119 | non-progressor | 25.1 | 6.1 | 16,475,534 | 5,363,274 | 1,790,966 |
| 120 | non-progressor | 24.9 | 6.1 | 17,912,774 | 5,929,224 | 489,731 |
| 121 | non-progressor | 26.1 | 6.3 | 16,388,029 | 6,029,320 | 964,492 |
| 122 | non-progressor | 26.1 | 6.1 | 17,750,716 | 7,929,721 | 2,206,092 |
| 123 | non-progressor | 27.1 | 5.9 | 9,142,864 | 4,333,324 | 1,269,961 |
| 124 | non-progressor | 33.2 | 6 | 25,682,499 | 3,917,421 | 428,337 |
| 125 | non-progressor | 24.8 | 6 | 21,652,375 | 5,078,495 | 631,609 |
| 126 | non-progressor | 25.8 | 6.7 | 22,753,470 | 6,863,757 | 704,022 |
| 127 | non-progressor | 30.1 | 5.8 | 9,756,846 | 5,262,392 | 1,207,276 |
| 128 | non-progressor | 28.7 | 5.9 | 33,217,996 | 9,331,851 | 1,014,133 |
| 129 | non-progressor | 30 | 6.3 | 6,185,474 | 2,044,094 | 470,853 |
| 130 | non-progressor | 22.6 | 6.2 | 7,184,136 | 3,447,730 | 940,494 |
| 131 | non-progressor | 25.6 | 6.7 | 1,482,754 | 722,751 | 197,011 |
| 132 | non-progressor | 28.4 | 6.1 | 635,691 | 318,017 | 107,139 |
| 133 | non-progressor | 27 | 6 | 10,597,982 | 4,569,081 | 2,111,013 |
| 134 | non-progressor | 24.4 | 6.7 | 9,982,829 | 4,077,844 | 2,314,627 |
| 135 | non-progressor | 29.4 | 5.9 | 6,882,204 | 3,992,657 | 2,055,568 |
| 136 | non-progressor | 27.6 | 5.7 | 22,090,746 | 2,421,874 | 482,182 |
| 137 | non-progressor | 32.9 | 5.8 | 8,308,149 | 5,793,961 | 2,346,521 |
| 138 | non-progressor | 26.7 | 5.8 | 11,675,494 | 6,822,226 | 4,679,696 |
| 139 | non-progressor | 27.4 | 6 | 9,131,519 | 5,469,710 | 3,208,117 |
| 140 | non-progressor | 23 | 5.6 | 24,754,286 | 2,457,131 | 67,158 |
| 141 | non-progressor | 24.2 | 6.3 | 8,890,963 | 7,476,495 | 2,188,559 |
| 142 | non-progressor | 21.9 | 5.7 | 6,731,691 | 5,579,104 | 2,033,151 |
| 143 | non-progressor | 28.2 | 6.5 | 16,990,625 | 7,884,100 | 5,788,171 |
| 144 | non-progressor | 26.3 | 5.7 | 13,301,031 | 7,462,321 | 3,169,669 |
| 145 | non-progressor | 32.8 | 6 | 4,828,102 | 1,458,738 | 613,262 |
| 146 | progressor | 25.8 | 5.7 | 11,645,654 | 5,012,172 | 2,867,927 |
| 147 | progressor | 29.4 | 6.3 | 12,865,406 | 8,445,621 | 4,985,428 |
| 148 | progressor | 27.3 | 5.8 | 7,176,592 | 3,383,580 | 1,524,903 |
| 149 | progressor | 29.2 | 5.9 | 7,449,947 | 3,915,162 | 2,486,897 |
| 150 | progressor | 32.3 | 6.7 | 4,075,043 | 3,476,413 | 1,994,373 |
| 151 | progressor | 23.2 | 6.2 | 5,814,552 | 2,992,581 | 1,573,692 |
| 152 | progressor | 25 | 5.9 | 94,116,726 | 74,520,225 | 43,804,576 |
| 153 | progressor | 24.4 | 5.6 | 16,010,877 | 11,445,377 | 4,077,499 |
| 154 | progressor | 30.8 | 6.2 | 12,152,458 | 10,345,691 | 5,861,364 |
| 155 | progressor | 27.4 | 6.8 | 16,023,300 | 3,299,510 | 934,312 |
| 156 | progressor | 23.6 | 5.8 | 7,507,473 | 5,395,881 | 2,186,441 |
| 157 | progressor | 27.9 | 5.6 | 16,190,975 | 2,013,719 | 736,529 |
| 158 | progressor | 27.3 | 5.6 | 6,201,950 | 2,725,715 | 1,474,979 |
| 159 | progressor | 23 | 5.9 | 7,756,683 | 6,682,453 | 2,866,443 |
| 160 | progressor | 33 | 6.4 | 6,695,930 | 4,319,660 | 2,744,557 |
| 161 | progressor | 29.9 | 5.7 | 10,759,783 | 7,537,355 | 4,121,651 |
| 162 | progressor | 29.7 | 5.6 | 13,422,051 | 6,955,859 | 3,411,592 |
| 163 | progressor | 32.9 | 6 | 7,485,657 | 6,824,156 | 3,227,831 |
| 164 | progressor | 25.9 | 6.4 | 11,036,010 | 3,174,708 | 1,337,345 |
| 165 | progressor | 22.2 | 6.5 | 9,012,136 | 4,453,094 | 2,144,199 |
| 166 | progressor | 29.9 | 6.7 | 12,487,053 | 3,691,940 | 1,595,685 |
| 167 | progressor | 30.2 | 6.1 | 15,003,722 | 3,326,957 | 908,624 |
| 168 | progressor | 30.6 | 6.3 | 8,317,556 | 3,568,309 | 1,648,085 |
| 169 | progressor | 28.4 | 6.2 | 10,224,913 | 3,947,765 | 2,290,923 |
| 170 | progressor | 33.2 | 6.4 | 7,238,314 | 3,810,865 | 1,875,047 |
| 171 | progressor | 30.1 | 5.7 | 13,026,845 | 3,252,582 | 1,331,154 |
| 172 | progressor | 30.5 | 5.9 | 15,718,078 | 4,287,799 | 1,301,561 |
| 173 | progressor | 30.6 | 5.9 | 22,023,198 | 808,851 | 254,468 |
| 174 | progressor | 23.6 | 6.7 | 32,994,138 | 3,393,710 | 267,058 |
| 175 | progressor | 23.7 | 6.6 | 8,887,967 | 5,523,546 | 2,652,981 |
| 176 | progressor | 27.2 | 6.4 | 14,118,063 | 1,425,949 | 208,290 |
| 177 | progressor | 28.4 | 6.1 | 9,008,660 | 5,054,419 | 2,024,108 |
| 178 | progressor | 22.4 | 6.4 | 7,316,009 | 3,847,167 | 2,164,524 |
| 179 | progressor | 26.5 | 6.7 | 9,257,702 | 3,656,088 | 1,486,668 |
| 180 | progressor | 33.1 | 6.3 | 6,461,865 | 2,876,152 | 1,360,359 |
| 181 | progressor | 25.2 | 6.6 | 17,909,054 | 12,734,437 | 2,630,828 |
| 182 | progressor | 32.2 | 6.4 | 7,392,377 | 3,433,406 | 963,467 |
| 183 | progressor | 31.1 | 6.7 | 9,178,095 | 3,570,557 | 1,601,818 |
| 184 | progressor | 28.1 | 6.6 | 10,593,297 | 7,027,565 | 4,411,223 |
| 185 | progressor | 24.8 | 5.8 | 12,053,861 | 8,695,433 | 5,176,073 |
| 186 | progressor | 28.2 | 6.9 | 9,166,567 | 5,141,990 | 2,342,791 |
| 187 | progressor | 26.2 | 5.7 | 7,361,274 | 5,885,277 | 2,629,136 |
| 188 | progressor | 26.8 | 5.8 | 6,972,010 | 4,674,905 | 1,857,421 |
| 189 | progressor | 28.9 | 6.6 | 8,209,623 | 3,933,339 | 2,731,536 |
| 190 | progressor | 24.2 | 5.7 | 9,305,435 | 6,069,926 | 2,664,653 |
| 191 | progressor | 27.1 | 5.7 | 14,568,000 | 2,183,435 | 303,410 |
| 192 | progressor | 22.1 | 6.2 | 23,888,938 | 7,758,209 | 1,611,155 |
| 193 | progressor | 28.9 | 5.8 | 18,611,225 | 2,095,183 | 292,903 |
| 194 | progressor | 25.2 | 6.2 | 18,012,951 | 2,109,204 | 433,307 |
| 195 | progressor | 29.7 | 6 | 17,993,170 | 752,856 | 119,245 |
| 196 | progressor | 28.1 | 6.4 | 12,694,539 | 1,393,232 | 195,786 |
| 197 | progressor | 25.9 | 5.8 | 19,115,427 | 2,686,181 | 543,414 |
| 198 | progressor | 27.5 | 6.7 | 10,762,503 | 3,857,702 | 887,488 |
| 199 | progressor | 31.8 | 6.4 | 10,510,542 | 3,920,999 | 1,390,118 |
| 200 | progressor | 37.8 | 6.3 | 11,390,569 | 2,135,909 | 628,948 |
| 201 | progressor | 31 | 5.8 | 14,476,649 | 2,269,080 | 672,818 |
| 202 | progressor | 28.9 | 6 | 7,816,807 | 4,428,001 | 1,576,769 |
| 203 | progressor | 25.2 | 6.1 | 7,660,432 | 6,345,169 | 1,957,026 |
| 204 | progressor | 34.3 | 6.1 | 12,910,507 | 3,210,309 | 774,788 |
| 205 | progressor | 28.7 | 6.3 | 10,103,471 | 8,092,013 | 4,540,243 |
| 206 | progressor | 26.8 | 6.3 | 6,982,310 | 2,997,746 | 1,369,530 |
| 207 | progressor | 25.1 | 6.3 | 14,323,187 | 7,525,012 | 3,270,599 |
| 208 | progressor | 32.5 | 6.5 | 9,764,731 | 3,785,652 | 1,983,985 |
| 209 | progressor | 24.8 | 5.7 | 10,673,544 | 4,456,802 | 2,084,039 |
| 210 | progressor | 24.3 | 6.7 | 8,895,372 | 1,582,845 | 679,435 |
| 211 | progressor | 32.6 | 6.1 | 10,037,655 | 3,149,317 | 1,272,603 |
| 212 | progressor | 34.6 | 6.3 | 9,976,801 | 3,540,199 | 2,037,329 |
| 213 | progressor | 26 | 6.9 | 15,194,964 | 8,440,689 | 4,972,414 |
| 214 | progressor | 28.2 | 6.1 | 12,395,882 | 576,444 | 134,135 |
| 215 | progressor | 33.6 | 6.9 | 18,447,869 | 3,518,203 | 1,124,038 |
| 216 | progressor | 27.9 | 6.4 | 8,931,709 | 1,905,418 | 762,957 |
| 217 | progressor | 26 | 6.4 | 8,381,685 | 7,105,063 | 5,474,451 |
| 218 | progressor | 30.2 | 5.7 | 11,173,063 | 3,423,260 | 1,726,936 |
| 219 | progressor | 30.3 | 5.9 | 11,561,732 | 6,102,883 | 3,218,584 |
| 220 | progressor | 26.1 | 6.7 | 7,892,982 | 5,644,121 | 1,903,083 |
| 221 | progressor | 26.2 | 6.3 | 6,099,659 | 3,983,688 | 2,195,086 |
| 222 | progressor | 29.2 | 6.6 | 12,072,291 | 6,932,421 | 3,883,689 |
| 223 | progressor | 26.8 | 5.8 | 9,860,155 | 4,604,995 | 1,708,574 |
| 224 | progressor | 32.6 | 5.6 | 7,775,802 | 3,153,574 | 1,501,893 |
| 225 | progressor | 30.9 | 6.2 | 11,605,424 | 7,988,431 | 2,461,305 |
| 226 | progressor | 30 | 6.1 | 8,315,781 | 4,216,305 | 2,194,672 |
| 227 | progressor | 22 | 6.1 | 8,730,790 | 6,083,942 | 2,054,534 |
| 228 | progressor | 27.5 | 6.5 | 7,980,124 | 4,947,204 | 2,617,141 |
| 229 | progressor | 33.1 | 5.9 | 9,711,553 | 7,239,323 | 4,613,264 |
| 230 | progressor | 28.7 | 6.6 | 11,928,545 | 5,813,826 | 3,378,990 |
| 231 | progressor | 24.7 | 5.9 | 7,728,396 | 3,722,957 | 1,630,513 |
| 232 | progressor | 25.4 | 5.9 | 7,930,722 | 5,162,782 | 2,475,236 |
| 233 | progressor | 23.8 | 6 | 10,892,806 | 4,628,461 | 1,776,167 |
| 234 | progressor | 27.3 | 6.2 | 7,547,028 | 1,566,930 | 720,658 |
| 235 | progressor | 35.8 | 6.8 | 12,626,118 | 7,954,296 | 2,194,035 |
| 236 | progressor | 29.1 | 5.9 | 21,292,663 | 6,094,055 | 1,707,899 |
| 237 | progressor | 26.9 | 6.9 | 15,410,473 | 10,568,733 | 5,598,755 |
| 238 | progressor | 24.5 | 5.6 | 13,082,092 | 5,721,779 | 2,511,205 |
| 239 | progressor | 29.4 | 6.2 | 9,142,587 | 6,397,076 | 3,101,073 |
| 240 | progressor | 33 | 6.1 | 6,871,158 | 5,544,256 | 3,122,361 |
| 241 | progressor | 28.8 | 6.2 | 7,416,434 | 6,282,504 | 3,437,244 |
| 242 | progressor | 28.2 | 6.4 | 6,998,532 | 4,781,193 | 2,189,855 |
| 243 | progressor | 26.9 | 6.1 | 8,110,385 | 3,416,600 | 1,346,609 |
| 244 | progressor | 36.1 | 6 | 9,391,305 | 7,728,839 | 2,953,484 |
| 245 | progressor | 28.6 | 6.1 | 10,914,593 | 3,010,105 | 1,048,123 |
| 246 | progressor | 26.6 | 5.6 | 10,057,609 | 6,082,822 | 2,957,796 |
| 247 | progressor | 22.5 | 6.5 | 36,113,367 | 29,490,163 | 11,617,079 |
| 248 | progressor | 25.3 | 6.7 | 12,981,501 | 8,449,389 | 3,370,115 |
| 249 | progressor | 25.7 | 6.5 | 8,171,094 | 6,571,953 | 4,009,159 |
| 250 | progressor | 30.4 | 6 | 9,187,970 | 1,368,678 | 480,962 |
| 251 | progressor | 28.9 | 6.4 | 13,315,648 | 2,101,552 | 606,260 |
| 252 | progressor | 27.1 | 6.4 | 6,656,264 | 3,485,164 | 1,273,867 |
| 253 | progressor | 24.9 | 6.1 | 8,032,072 | 5,582,469 | 2,576,665 |
| 254 | progressor | 29.7 | 6.7 | 13,953,582 | 4,516,316 | 1,436,149 |
| 255 | progressor | 27.6 | 6.6 | 16,128,749 | 13,530,903 | 6,289,640 |
| 256 | progressor | 27.8 | 6.2 | 8,982,955 | 7,032,372 | 4,034,770 |
| 257 | progressor | 29.6 | 5.9 | 16,392,432 | 5,460,155 | 2,147,059 |
| 258 | progressor | 23.4 | 5.9 | 8,509,625 | 3,926,670 | 2,142,456 |
| 259 | progressor | 31.5 | 6.5 | 4,934,065 | 2,501,140 | 1,062,138 |
| 260 | progressor | 26.9 | 6 | 12,396,720 | 6,174,883 | 2,594,044 |
| 261 | progressor | 28.9 | 6.7 | 7,188,214 | 4,635,047 | 2,377,900 |
| 262 | progressor | 26.6 | 6.8 | 13,136,225 | 11,304,852 | 6,159,617 |
| 263 | progressor | 30.1 | 6.2 | 8,943,876 | 5,383,181 | 2,698,089 |
| 264 | progressor | 29.4 | 6.6 | 30,395,195 | 20,048,363 | 9,596,660 |
| 265 | progressor | 26.7 | 6 | 11,544,286 | 7,868,645 | 4,499,876 |
| 266 | progressor | 29.4 | 5.8 | 7,309,785 | 3,645,101 | 1,683,002 |
| 267 | progressor | 32.4 | 6.2 | 6,062,322 | 4,177,441 | 2,021,984 |
| 268 | progressor | 31.5 | 6 | 21,868,207 | 4,317,699 | 1,167,973 |
| 269 | progressor | 27.5 | 6.3 | 25,912,711 | 3,743,519 | 697,715 |
| 270 | progressor | 27.5 | 6 | 13,177,323 | 2,832,016 | 699,437 |
| 271 | progressor | 29.6 | 5.7 | 12,711,748 | 4,268,460 | 1,032,066 |
| 272 | progressor | 31.2 | 6.6 | 26,864,171 | 4,252,575 | 1,232,203 |
| 273 | progressor | 23.1 | 6.5 | 13,061,457 | 3,340,367 | 647,234 |
| 274 | progressor | 32 | 6.1 | 13,901,706 | 3,614,633 | 464,699 |
| 275 | progressor | 23.3 | 6 | 19,610,847 | 7,142,477 | 500,589 |
| 276 | progressor | 28.2 | 6.2 | 12,753,600 | 9,024,973 | 2,717,777 |
| 277 | progressor | 21.2 | 5.6 | 15,422,143 | 4,773,163 | 816,370 |
| 278 | progressor | 27.7 | 6.9 | 27,112,366 | 9,753,333 | 2,010,645 |
| 279 | progressor | 26.8 | 6.5 | 14,633,283 | 3,343,070 | 394,755 |
| 280 | progressor | 28.1 | 6.2 | 10,233,196 | 5,634,120 | 1,284,918 |
| 281 | progressor | 24.8 | 6.8 | 14,462,497 | 1,999,270 | 457,960 |
| 282 | progressor | 28.2 | 6.2 | 19,533,033 | 3,574,906 | 598,435 |
| 283 | progressor | 34.1 | 5.8 | 25,807,708 | 11,303,689 | 1,598,738 |
| 284 | progressor | 27.3 | 6.1 | 6,545,931 | 4,013,192 | 1,494,343 |
| 285 | progressor | 33.7 | 6.9 | 9,446,360 | 5,522,949 | 1,755,528 |
| 286 | progressor | 32.8 | 6.4 | 7,091,309 | 4,034,607 | 1,779,474 |
| 287 | progressor | 24.3 | 6.8 | 8,152,655 | 4,486,860 | 2,260,019 |
| 288 | progressor | 32.3 | 5.9 | 9,746,162 | 4,970,847 | 2,263,218 |
| 289 | progressor | 27.1 | 6.5 | 7,020,126 | 4,416,832 | 2,269,526 |
| 290 | progressor | 32.8 | 6.6 | 10,106,244 | 4,774,768 | 2,789,869 |

**Supplemental Table 2.** Multivariate analysis of covariance (MANCOVA) t-test results from miRNAs that show concertation differences between progressors and non-progressors.

| miRNA | Log2 Fold Change | MANOCOVA |
| --- | --- | --- |
| hsa-miR-122-5p | 0.66 | 0.001303 |
| hsa-miR-376b-3p | 0.66 | 0.012464 |
| hsa-miR-4532-5p | 0.66 | 0.004906 |
| hsa-miR-660-3p | 0.63 | 0.000116 |
| hsa-miR-378a-3p | 0.62 | 0.001832 |
| hsa-miR-3200-3p | 0.61 | 0.000559 |
| hsa-miR-210-3p | 0.61 | 0.004204 |
| hsa-miR-483-5p | 0.59 | 0.005191 |
| hsa-miR-550a-1-3p | 0.55 | 0.000962 |
| hsa-miR-190a-5p | 0.53 | 0.000481 |
| hsa-miR-192-5p | 0.52 | 0.002851 |
| hsa-miR-193b-3p | 0.52 | 0.004497 |
| hsa-miR-127-5p | 0.51 | 0.009518 |
| hsa-miR-6087-3p | 0.51 | 0.001843 |
| hsa-miR-99a-5p | 0.50 | 0.00624 |
| hsa-miR-145-5p | 0.48 | 0.014 |
| hsa-miR-193a-5p | 0.48 | 0.006915 |
| hsa-miR-6087-5p | 0.47 | 0.007583 |
| hsa-miR-100-5p | 0.46 | 0.008488 |
| hsa-miR-505-3p | 0.46 | 0.008628 |
| hsa-miR-29a-5p | 0.45 | 0.001314 |
| hsa-miR-150-5p | 0.45 | 0.004589 |
| hsa-miR-29c-5p | 0.45 | 0.013085 |
| hsa-miR-10b-5p | 0.45 | 0.009209 |
| hsa-miR-17-3p | 0.44 | 0.046962 |
| hsa-miR-106b-3p | 0.43 | 0.010291 |
| hsa-miR-627-5p | 0.42 | 0.038185 |
| hsa-miR-487b-3p | 0.40 | 0.041124 |
| hsa-miR-134-5p | 0.40 | 0.047239 |
| hsa-miR-144-3p | 0.39 | 0.025812 |
| hsa-miR-24-2-5p | 0.39 | 0.03898 |
| hsa-miR-181a-1-3p | 0.39 | 0.019734 |
| hsa-miR-320a-3p | 0.38 | 0.032595 |
| hsa-miR-7641-1-3p | 0.37 | 0.011865 |
| hsa-miR-181b-2-5p | 0.36 | 0.020694 |
| hsa-miR-10b-3p | 0.35 | 0.027194 |
| hsa-miR-370-3p | 0.35 | 0.038899 |
| hsa-miR-7-1-5p | 0.34 | 0.009094 |
| hsa-miR-215-5p | 0.33 | 0.033258 |
| hsa-miR-320b-1-3p | 0.33 | 0.041752 |
| hsa-miR-874-3p | 0.32 | 0.043494 |
| hsa-miR-1246-5p | 0.32 | 0.044871 |
| hsa-miR-10a-5p | 0.32 | 0.04845 |
| hsa-miR-3615-3p | 0.31 | 0.026191 |
| hsa-miR-375-3p | 0.31 | 0.045195 |
| hsa-miR-500a-3p | 0.30 | 0.042999 |
| hsa-miR-125b-1-5p | 0.30 | 0.039774 |
| hsa-miR-34a-5p | 0.29 | 0.043599 |
| hsa-miR-30a-3p | 0.35 | 0.073492 |
| hsa-miR-451a-5p | 0.34 | 0.051774 |
| hsa-miR-483-3p | 0.34 | 0.050438 |
| hsa-miR-194-1-5p | 0.33 | 0.090042 |
| hsa-miR-150-3p | 0.33 | 0.059675 |
| hsa-miR-101-1-5p | 0.33 | 0.06331 |
| hsa-miR-1228-5p | 0.31 | 0.053276 |
| hsa-miR-30a-5p | 0.28 | 0.066195 |
| hsa-miR-486-1-5p | 0.28 | 0.059636 |

Underlined results are non-significant miRNAs (P>0.05)

**Supplemental Table 3.** List of novel miRNA candidates showing concentration changes in the study.

| **miRNA ID** | **sequence** | Progressor  /   Non-progressor | |  | Progressor | | | Non-progressor | | |  | | Normal (BMI <25) | | | | Obese (BMI>30) | |  |  |
| --- | --- | --- | --- | --- | --- | --- | --- | --- | --- | --- | --- | --- | --- | --- | --- | --- | --- | --- | --- | --- |
|  |  |  |  |  | **Obese (BMI>30)/Normal (BMI<25)** | | | | | |  | | **Progressor/Non-progressor** | | | | | | | |
|  |  | Log 2 FC* | p-value |  | | Log 2 FC (PO vs PN) | p-value | | Log 2 FC (NPO vs NPN) | p-value | |  | | Log 2 FC (PN vs NPN) | p-value | Log 2 FC (PO vs. NPO) | | p-value | |  |
| novel:chr01_109-3p | GUAACUAGGAUAACUGUGGUAA | 0.51 | 0.0044 |  | |  |  | |  |  | |  | |  |  | 0.87 | | 0.0145 | |  |
| novel:chr01_129-5p | UGCAGUCUAGUGGUUAGGAUUC | 0.46 | 0.0293 |  | |  |  | |  |  | |  | |  |  | 0.89 | | 0.0369 | |  |
| novel:chr02_1722-5p | GUGGAAUGAGUACAAUUUAACU |  |  |  | | -0.69 | 0.0328 | |  |  | |  | |  |  |  | |  | |  |
| novel:chr04_2188-5p | UCCCGGGUUUCGGCACCAGAUG | 0.38 | 0.0133 |  | |  |  | |  |  | |  | |  |  |  | |  | |  |
| novel:chr05_2330-5p | GAUUUUUGGAAAUAGGAGUGUA | **0.67** | **0.0013** |  | |  |  | |  |  | |  | |  |  |  | |  | |  |
| novel:chr06_2537-3p | UAGUCCCUGUUCGGGCGCCACU | 0.42 | 0.0005 |  | |  |  | |  |  | |  | |  |  | 0.55 | | 0.0357 | |  |
| novel:chr08_2805-3p | UUACUACUGCACUUGACUAGUC | 0.30 | 0.0281 |  | |  |  | |  |  | |  | |  |  |  | |  | |  |
| novel:chr08_2814-3p | AAGGGGCGGGGCGAAGCCAGAG | 0.40 | 0.0238 |  | |  |  | |  |  | |  | |  |  |  | |  | |  |
| novel:chr09_2875-5p | CGAGGGCGCGCGGGUCGGUUCU |  |  |  | |  |  | |  |  | |  | |  |  | 0.89 | | 0.02685 | |  |
| novel:chr09_2888-5p | AAGCGGAGGAGAAGAAACCAGA | 0.47 | 0.0116 |  | |  |  | |  |  | |  | |  |  | 0.69 | | 0.0403 | |  |
| novel:chr10_296-5p | GAUGGAGUGGAGCCUGCGGCCA |  |  |  | | 0.75 | 0.0198 | |  |  | |  | |  |  |  | |  | |  |
| novel:chr14_793-3p | CAUAAAAAGUUGAAAAGAACUU | 0.47 | 0.0071 |  | | 1.09 | 0.0044 | |  |  | |  | |  |  | 0.69 | | 0.0440 | |  |
| novel:chr15_963-3p | GGAAAUAAUUUGUGGUAGUGGG | 0.34 | 0.0252 |  | | -0.66 | 0.0318 | |  |  | |  | | 0.92 | 0.0091 |  | |  | |  |
| novel:chr16_1043-3p | AAAUAAACAGAAAAUUCUGAGU | **0.68** | **0.0009** |  | |  |  | |  |  | |  | |  |  | 0.87 | | 0.0373 | |  |
| novel:chr18_1321-5p | GAAGAGACAUGAGAGGAGACUU | **0.63** | **0.0037** |  | |  |  | |  |  | |  | |  |  | 1.04 | | 0.0127 | |  |
| novel:chr20_1748-3p | UGCCCGGCGGUGUGCGGCCACA | 0.36 | 0.0123 |  | |  |  | |  |  | |  | |  |  | 0.54 | | 0.0412 | |  |

PO: Progressors Obese, PN: progressors normal, NPO: non-progressors obese, NPN: non-progressors normal. Boldface character indicates greater than 1.5 fold concentration change.

**Supplemental Figure 1:** Bioanalyzer trace images from RNA extracted from plasma

Representative images taken from 75 uL of (A) progressor and (B) non-progressor patient plasma samples

**Supplemental Figure 2:** Differing normalization methods on miRNAs followed up on this study. We compared the normalization method used in this study (log2RPM using processed reads – blue) to two other alternative methods (log2RPM using miRNA reads – red, and Limma/vroom - green). We looked at the miRNAs that were followed up and highlighted in this study (miR-122-5p, miR-376b-3p, miR-3200-3p, miR-210-3p, miR-192-5p, miR-127-5p, miR-99a-5p, miR-29a-5p, miR-4532-5p, miR-378a-3p, miR-483-5p, mir-375-3p, miR-7641-1-3p, and miR-660-3p, and found that 11/14 had similar significant (P <0.05) fold changes (>1.2) between all three comparisons.

**Supplemental Figure 3:** Comparison of miRNA panel performance to threshold FPG.

The classification performance (AUC values from ROC analysis) of our two miRNA panels (5 miRNA panel: miR-660-3p, miR-3200-3p, miR-4532-5p, miR-122-5p, and miR-378a-3p; 8 miRNA panel: miR-7641-3p, miR-136-5p, miR-490-3p, miR-501-5p, miR-127-5p, miR-4532-5p, miR-483-5p, miR-210-3p) and the performance of threshold FPG alone. The AUC values for all patients (black bars), normal BMI patients (brown bars), and obese BMI patients (red bars) is shown for all results.
