# Supplementary figures and images for: Circulating RNAs as predictive markers for the progression of type 2 diabetes
Source: J Cell Mol Med. 2019 Feb 7;23(4):2753–68. doi: 10.1111/jcmm.14182 (PMC6433655; doi:10.1111/jcmm.14182)

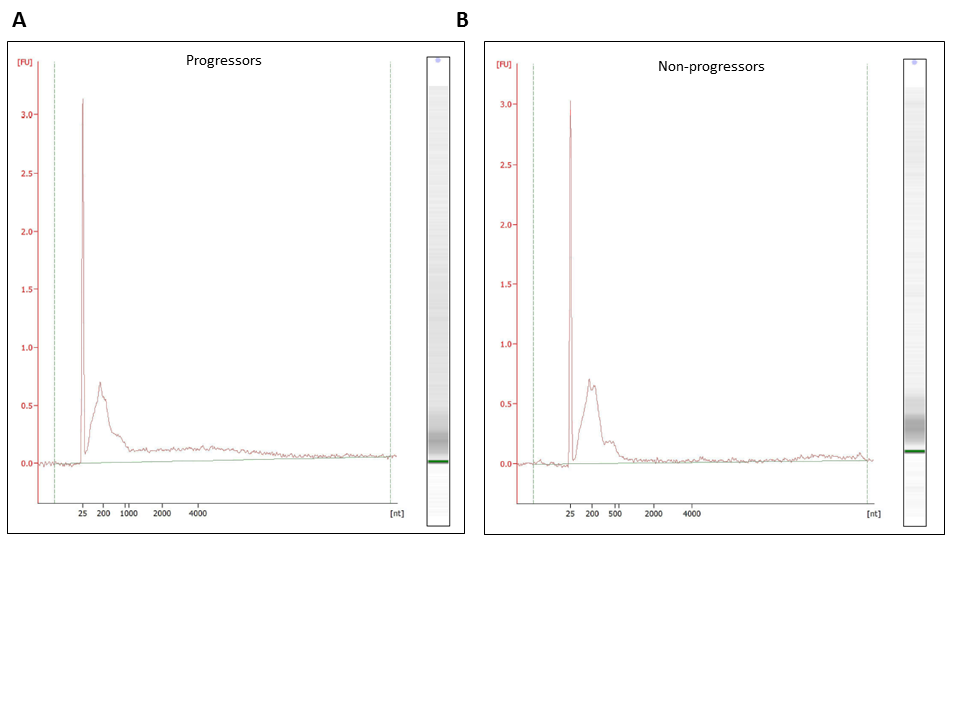

Supplement: Supplementary file 2 [file JCMM-23-2753-s002.TIF]

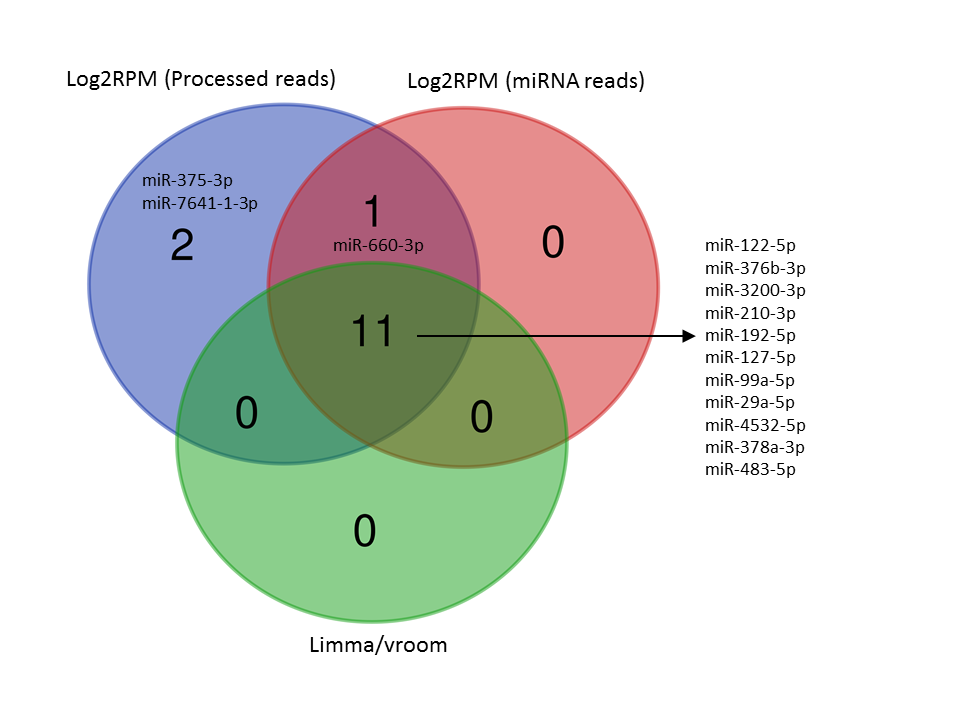

Supplement: Supplementary file 3 [file JCMM-23-2753-s003.TIF]

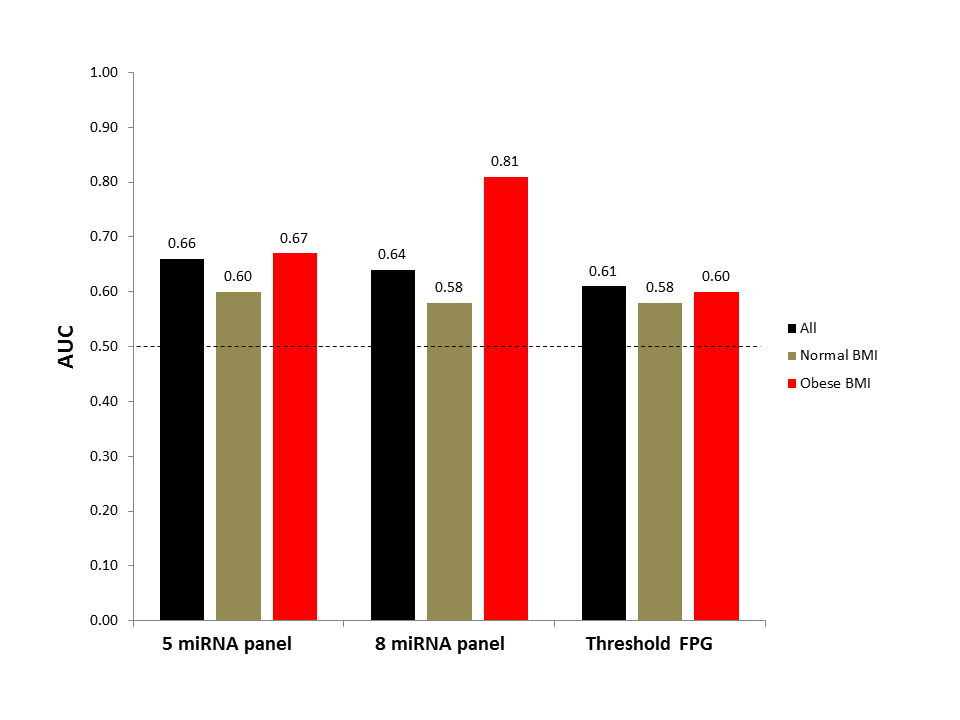

Supplement: Supplementary file 4 [file JCMM-23-2753-s004.TIF]
